# Modeling embryo-endometrial interface recapitulating human embryo implantation

**DOI:** 10.1126/sciadv.adi4819

**Published:** 2024-02-23

**Authors:** Shun Shibata, Shun Endo, Luis A. E. Nagai, Eri H. Kobayashi, Akira Oike, Norio Kobayashi, Akane Kitamura, Takeshi Hori, Yuji Nashimoto, Ryuichiro Nakato, Hirotaka Hamada, Hirokazu Kaji, Chie Kikutake, Mikita Suyama, Masatoshi Saito, Nobuo Yaegashi, Hiroaki Okae, Takahiro Arima

**Affiliations:** ^1^ Department of Informative Genetics, Tohoku University Graduate School of Medicine, Sendai 980-8575, Japan.; ^2^ Research and Development Division, Rohto Pharmaceutical Co. Ltd., Osaka 544-8666, Japan.; ^3^ Department of Obstetrics and Gynecology, Tohoku University Graduate School of Medicine, Sendai 980-8575, Japan.; ^4^ Laboratory of Computational Genomics, Institute for Quantitative Biosciences, The University of Tokyo, Tokyo 113-0032, Japan.; ^5^ Department of Trophoblast Research, Institute of Molecular Embryology and Genetics, Kumamoto University, Kumamoto 862-0973, Japan.; ^6^ Department of Mechanical Engineering, University of Michigan, Ann Arbor, MI, USA.; ^7^ Department of Diagnostic and Therapeutic Systems Engineering, Institute of Biomaterials and Bioengineering, Tokyo Medical and Dental University, Tokyo 101-0062, Japan.; ^8^ Division of Bioinformatics, Medical Institute of Bioregulation, Kyushu University, Fukuoka 812-8582, Japan.

## Abstract

The initiation of human pregnancy is marked by the implantation of an embryo into the uterine environment; however, the underlying mechanisms remain largely elusive. To address this knowledge gap, we developed hormone-responsive endometrial organoids (EMO), termed apical-out (AO)–EMO, which emulate the in vivo architecture of endometrial tissue. The AO-EMO comprise an exposed apical epithelium surface, dense stromal cells, and a self-formed endothelial network. When cocultured with human embryonic stem cell–derived blastoids, the three-dimensional feto-maternal assembloid system recapitulates critical implantation stages, including apposition, adhesion, and invasion. Endometrial epithelial cells were subsequently disrupted by syncytial cells, which invade and fuse with endometrial stromal cells. We validated this fusion of syncytiotrophoblasts and stromal cells using human blastocysts. Our model provides a foundation for investigating embryo implantation and feto-maternal interactions, offering valuable insights for advancing reproductive medicine.

## Teaser

Advanced 3D model mimics human embryo implantation at the embryo-endometrium interface.

## INTRODUCTION

The implantation process represents the initial phase of physical interaction between the developing embryo and the maternal endometrium. A blastocyst hatches from the zona pellucida, exposing the trophectoderm (TE), the main point of contact with the receptive endometrium, thus giving rise to the placenta and facilitating the exchange of nutrients, gas, and waste. In mice, embryos are positioned in crypts that branch off the antimesometrial uterine lumen, attach to the uterine wall with their inner cell mass (ICM) facing the cavity, and penetrate the surrounding endometrial epithelial cells by inducing apoptosis or entosis (*1*). Although analyses in mice have provided valuable insights into the biological processes and molecular mechanisms underlying implantation, there are considerable differences in this process between mice and humans. Unlike in mice, human blastocysts implant with their ICM oriented toward the apical surface of the luminal epithelium and penetrate the endometrium deeply until they become fully embedded (*2*, *3*).

The human endometrium undergoes cyclic changes throughout the menstrual cycle, driven by hormonal fluctuations. Progesterone, produced by the corpus luteum, primes the uterus for implantation (*4*). The endometrial lining is most receptive to an implanting embryo during a limited period known as the “implantation window,” which typically spans 4 to 6 days during the mid-luteal phase (*5*). The endometrial tissue undergoes substantial modifications, leading up to this critical phase. Edema manifests in the superficial stroma and becomes more extensive as the implantation window approaches (*6*). The luminal epithelium, which separates the uterine cavity from the underlying stroma, experiences alterations in cell polarity. The expression of adherence junctions and adhesion molecules diminishes, facilitating embryo invasion by weakening lateral cell-cell and cell-matrix adhesion (*7*). Concurrently, the glandular epithelium transitions from a tubular to a coiled configuration, a change associated with increased secretory capacity (*8*). These changes are accompanied by the differentiation of human endometrial stromal cells (eSC) into specialized decidual stromal cells, which play key roles in successful pregnancy (*9*). Immune cells undergo notable changes, most prominently the uterine natural killer (uNK) cells. Their numbers surge after ovulation, and they assume specialized functions in early pregnancy, such as remodeling spiral arteries and facilitating trophoblast invasion and embryo growth (*9*, *10*). Progesterone also regulates the endometrium’s blood flow, increasing it during the secretory phase and decreasing it before menstruation (*11*).

Therefore, successful implantation requires synchronization between the acquisition of implantation competency by the blastocyst and the receptive state of the endometrium (*12*). However, the detailed process underlying the embryo-endometrial interface during human implantation remains largely unknown due to ethical and technical barriers to obtaining early human biological samples. Consequently, using in vitro models that mimic implantation serves as an ethical and effective alternative. However, these implantation models developed by using immortalized cell lines (*13*) or cancer cells (*14*) may not accurately represent normal physiological conditions. Furthermore, the utilization of implantation models developed using human embryos (*15*, *16*) is restricted because of ethical considerations. Recent studies have highlighted the potential of human embryonic stem cells (ESC) to form TE and primitive endoderm lineages (*17*–*19*) and generate blastocyst-like structures called “blastoid” (*20*–*22*). However, their use in implantation is limited to two-dimensional (2D) culture.

In terms of the maternal models, advances in organoid culture techniques have enabled the long-term maintenance of human endometrial epithelial cells that are responsive to hormonal cues (*23*, *24*). These techniques enable detailed investigations of various conditions, including endometriosis or cancer (*25*), and analysis of epithelial-stromal interactions (*16*). 3D endometrial models constructed using these organoids and primary cells are promising tools for simulating physiological implantation (*26*). However, the cystic structure and reversed apicobasal polarity of conventional endometrial organoids (EMO) limit their utility in studying interactions with embryo surrogates during implantation (*4*).

In this study, we developed an apical-out (AO)–EMO, which exposes the apical surface of the epithelium and contains eSC and an endothelial network. By coculturing with human blastoids, we generated feto-maternal assembloids that recapitulated the initial stages of implantation, including apposition, adhesion, and invasion. This model enabled us to observe the direct interaction between fetal and maternal cells; it revealed that syncytia disrupt the endometrial epithelial barrier, invade human eSC, and fuse with them. This phenomenon was further confirmed by culturing human embryos. The feto-maternal assembloids presented in this study provide a valuable platform for visualizing the moment of implantation, analyzing the implantation mechanism, and developing fertility treatments.

## RESULTS

### Generation of AO-EMO

**Fig. 1.**
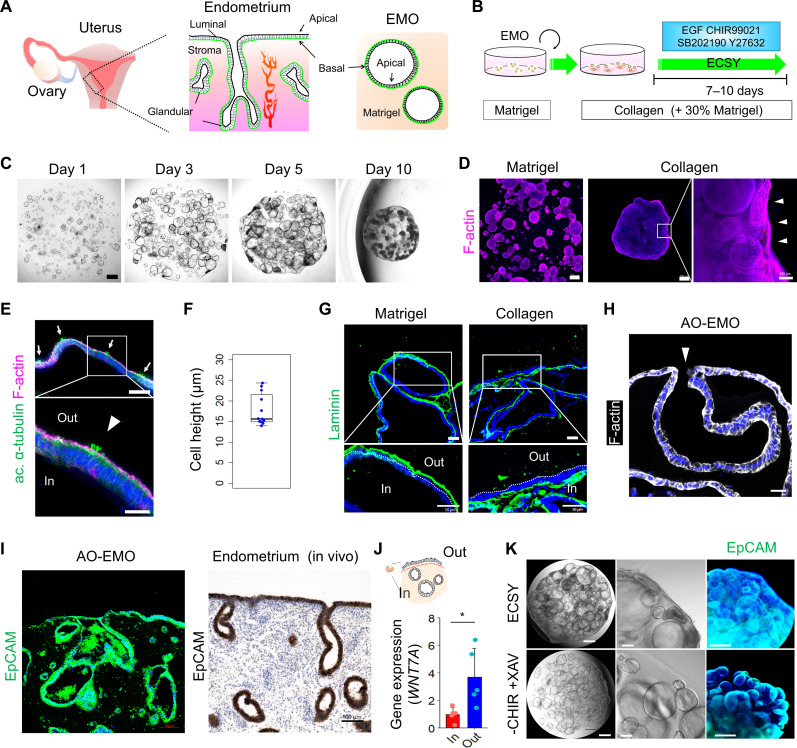
Generation of AO-EMO. (**A**) Schematic illustration of the anatomical structure of the endometrium alongside a representation of endometrial organoids; the basal membrane is highlighted in green. (**B**) Schematic representation of the culture method. (**C**) Bright-field images of EMO in a collagen-based gel on days 1, 3, 5, 7, and 10. Scale bar, 500 μm. (**D**) Whole-mount imaging of EMO cultured in Matrigel and a collagen-based gel stained for F-actin; nuclei stained with Hoechst (blue). Arrowheads indicate epithelial cells lining the surface of the gel. Scale bars, 500 μm and 100 μm (magnified). (**E**) Fluorescence images of AO-EMO stained for acetylated (ac.) α-tubulin and F-actin; nuclei stained with Hoechst (blue). Scale bars, 100 μm. (**F**) Quantifying of surface epithelial cell height of AO-EMO; *n* = 12 sections from six individual organoids. (**G**) Immunostaining images of EMO cultured in Matrigel and collagen-based gel stained for laminin; nuclei stained with Hoechst (blue). Scale bars, 50 μm. (**H**) Fluorescence image of AO-EMO stained for F-actin; nuclei stained with Hoechst (blue); the arrowhead indicates an open-up region. Scale bar, 20 μm. (**I**) Immunostaining image of AO-EMO stained for EpCAM; nuclei are stained with Hoechst (blue) (left). Immunohistochemistry of human endometrial tissue stained for EpCAM, and nuclei stained with hematoxylin (right). Scale bars, 100 μm. (**J**) Relative gene expression levels of *WNT7A* in the outer (Out) and inner (In) cells in AO-EMO (control). Data show means ± SD; *P < 0.05; *n* = 5 AO-EMO from four donors. (**K**) Bright-field and immunostaining images of EMO in collagen-based gels cultured with ECSY and ECSY minus CHIR99021 and plus XAV939. Scale bars, 500 μm (left) and 100 μm (center and right).

We used established techniques (*23*, *24*) to generate EMO from human endometrial and decidual tissues (Fig. 1A and fig. S1, A to C). Furthermore, we verified that EMO could be established and maintained in a simple medium supplemented with four factors: epidermal growth factor (EGF), CHIR99021, SB202190, and Y27632 (designated as ECSY). To better mimic the extracellular environment in vivo, we explored the addition of collagen to 3D culture substrates during EMO culture, given the high collagen content of endometrial tissue (Fig. 1B) (*27*, *28*). The use of collagen and subsequent ECSY culture stimulated the outward migration of epithelial cells and their exposure to the gel surface. Specifically, gel contraction was initiated at the sites where epithelial cells reached the periphery of the gel (fig. S1D). In contrast to Matrigel, where EMO were dispersed, the culture within the collagen-based gel resulted in the formation of a single aggregate (Fig. 1, C and D). The enhancement of EMO aggregation was directly proportional to the increase in collagen content in the matrix (fig. S1, E and F). On the basis of their morphology, we used a collagen-based gel that contained 70% type I collagen and 30% Matrigel in subsequent studies. The migrated epithelial cell layer on the gel surface was characterized by an F-actin and acetylated (ac.) α-tubulin^+^ (cilia marker) apical side facing outward (Fig. 1E). Upon measuring the length from basal to apical sides, the average cell height was found to be ~20 μm (mean, 17.8 μm), a value comparable to the typical height observed in the human endometrial luminal epithelial layer (Fig. 1F) (*29*). Immunostaining with laminin revealed an inverted apicobasal polarity of EMO cultured in collagen-based gel (Fig. 1G and fig. S1G). Therefore, we labeled these EMO as AO-EMO. The developed AO-EMO demonstrated a continuous connection between the surface and inner, flexed epithelial cells. In addition, some regions within the AO-EMO exhibited an “open-up” structure in which the inner epithelial cavities were connected to the external environment (Fig. 1H). This allows for the potential release of glandular secretions into the exterior. On average, each AO-EMO contained 2.36 open-up regions (fig. S1, H and I). These features demonstrate the spatial similarity between AO-EMO and in vivo endometrial tissue (Fig. 1I).

In 2D culture, the radial migration of EMO-derived epithelial cells was observed only in the presence of a collagen coating (fig. S1J). This finding indicates that the collagen matrix plays a crucial role in promoting the migration of endometrial epithelial cells, leading to the exposure of cells to the gel surface under 3D culture conditions.

Next, we examined the potential differences between the outer and inner epithelial cells in AO-EMO by manually pipetting the surface cells after trypsin treatment to distinguish between the two populations (fig. S1K). Gene expression analysis revealed that the outer cells exhibited increased expression of *WNT7A*, reflecting the surface epithelial characteristics of the endometrial tissue (Fig. 1J) (*30*). The outer cells also demonstrated a more dynamic state compared to the inner cells, with enhancement of the Gene Ontology (GO) terms “positive regulation of cell motility,” “positive regulation of locomotion,” and “positive regulation of cell migration,” reflecting a phenotype that migrates to cover the surface of gel (fig. S1L). The removal of CHIR99021 or the addition of the Wnt inhibitor XAV939 suppressed the AO trait observed on the gel surface, suggesting that the Wnt signaling is related to the formation of AO-EMO (Fig. 1K and fig. S1M).

### Collagen-based culture enhances maturation and spatial heterogeneity of endometrial epithelial cells

**Fig. 2.**
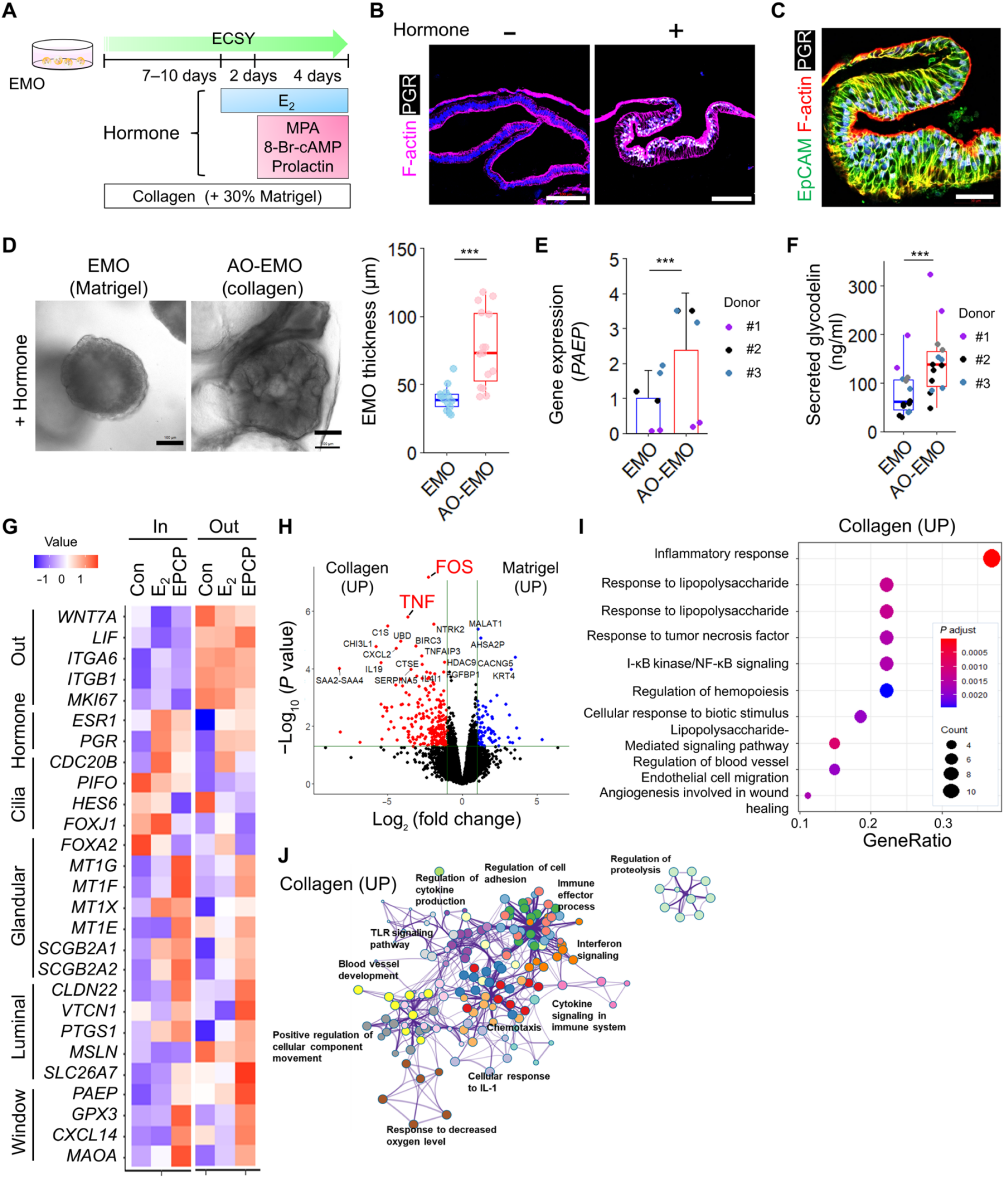
Collagen-based culture enhances the maturation and spatial heterogeneity of endometrial epithelial cells. (**A**) Schematic of the culture procedure. (**B**) Fluorescence images of AO-EMO with (+) or without (−) hormone (EPCP) stained for F-actin and PGR; nuclei stained with Hoechst (blue). Scale bars, 100 μm. (**C**) Fluorescence image of hormone-treated AO-EMO stained for EpCAM, F-actin, and PGR; nuclei stained with Hoechst (blue). Scale bar, 50 μm. (**D**) Bright-field images of EMO in Matrigel and collagen-based gel (left). Scale bars, 100 μm. Quantification of EMO thickness (right); *n* = 15 (collagen) and 13 (Matrigel) organoids. ***P < 0.001. (**E**) Relative gene expression levels of *PAEP* in EMO cultured in Matrigel and a collagen-based gel as measured by quantitative reverse transcriptase polymerase chain reaction (PCR). Data show means ± SD; ***P < 0.001; *n* = 6 EMO from three donors. (**F**) Quantification of secreted glycodelin (PAEP); *n* = 14 organoids; ***P < 0.001. (**G**) Heatmap of relative gene expression levels of the outer and inner cells of control (Con), E2-treated (E_2_), EPCP-treated (EPCP) AO-EMO as measured by RNA-seq. Data represent the means of scaled TPM from four AO-EMO derived from four donors. (**H**) Volcano plot of differentially expressed genes in EMO cultured in Matrigel versus EMO cultured in a collagen-based gel. Genes with an adjusted *P* value less than 0.05 and log_2_(fold change) > 1 are considered significantly differentially expressed. (**I**) GO terms related to biological processes enriched in up-regulated genes in EMO cultured in a collagen-based gel. (**J**) GO term network of up-regulated genes in EMO cultured in a collagen-based gel. TLR, Toll-like receptor. UP, up-regulated.

To investigate the hormonal response of AO-EMO, we initially added 17β-estradiol (E_2_) for a period of 2 days, followed by a combined addition of E_2_, medroxyprogesterone acetate (MPA), 8-bromoadenosine 3′,5′-cyclic monophosphate (8-Br-cAMP), and prolactin for an additional 4 days. These hormones are commonly used for the maturation of EMO (Fig. 2A) (*23*, *30*, *31*). Immunostaining results showed that the hormone addition increased the expression of progesterone receptors (PGRs). Furthermore, the morphology of AO-EMO adopted a coiled shape, similar to that observed in the endometrial glands during the secretary phase (Fig. 2, B and C) (*4*). When hormones were added to AO-EMO cultured within collagen-based gel, a marked thickening was observed compared to that in those cultured within Matrigel (Fig. 2D). In this collagen-based gel environment, AO-EMO showed elevated gene expression and levels of secreted glycodelin, also known as progestagen-associated endometrial protein (PAEP) (*32*), when compared to those in Matrigel (Fig. 2, E and F). This suggests that the collagen-based gel may enhance the hormonal responsiveness and maturity of EMO.

Next, we analyzed the transcriptome of each epithelial cell in the AO-EMO, which were divided into outer and inner cells following stepwise hormonal treatment. We conducted principal components analysis (PCA) to compare AO-EMO with conventional EMO (*31*) and endometrial epithelial cells in vivo (*33*). Plotting the data on principal component 1 (PC1) and PC2 divided the clusters into three groups while plotting those on PC2 and PC3 located the in vivo epithelial cells and AO-EMO in close proximity (fig. S2A). We extracted the PC1, PC2, and PC3 genes and performed GO analyses. For PC1, which distinguished between in vitro and in vivo epithelial cells, characteristic terms such as “multicellular organic homeostasis” and “anatomic structure homeostasis” were enriched. PC2, which separated conventional EMO from in vivo epithelial cells and AO-EMO, was enriched with terms such as “response to hypoxia” and “response to interleukin-1.” The enriched terms for genes in PC3 were related to hormonal response, such as “maternal process involved in female pregnancy” or “decidualization” (fig. S2B). Furthermore, the transcriptome analysis of the outer and inner cells of AO-EMO revealed distinct characteristics between them. The outer cells consistently showed high expression levels of *LIF*, an important factor for implantation (*34*), as well as integrins (*ITGA6* and *ITGB1*) involved in adhesion; and *MKI67*, a marker of the proliferative phase. In contrast, the inner cells were characterized by a high expression of hormone-responsive genes (*ESR1* and *PGR*) and ciliated epithelial markers (*CDC20B*, *PIFO*, *HES6*, and *FOXJ1*). The inner epithelial cells also consistently showed increased expression of genes, including *Metallothionein* (*MT*) and markers, such as *SCGB2A1* and *SCGB2A2*, expressed in the glandular epithelium in response to hormones (*30*). After hormone treatment, markers of luminal epithelial cells at the secretory phase (*CLDN22*, *VTCN1*, *PTGS1*, *MSLN*, and *SLC26A7*) (*30*) tended to be highly expressed in the outer cells. Genes up-regulated at the implantation window (*35*), including *PAEP*, *GPX3*, *CXCL14*, and *MAOA*, showed a gradual increase in expression after treatment (Fig. 2G). A pre-ranked gene set enrichment analysis (GSEA) was conducted on a group of genes that were up-regulated by hormonal treatment of the outer cells of the AO-EMO (fig. S2C). Among them, “complement and coagulation cascades” were also enriched in the up-regulated genes in the in vivo endometrium used for endometrium receptivity analysis (fig. S2D) (*35*, *36*). These findings further support that AO-EMO mature in response to hormones and suggest that AO-EMO exhibit a spatial heterogeneity, which is relatively similar to that observed in vivo.

To investigate the effects of different substrates on EMO, we compared the transcriptomes of EMO cultured within Matrigel and collagen-based gels before forming AO structures. An increase in the expression of genes related to the “inflammatory response,” such as *FOS* and *TNF*, was observed in the collagen-based culture (Fig. 2, H and I). Furthermore, the GO term network revealed that the collagen-based culture promoted the inflammation-related signaling networks and hypoxia, as the terms “cellular response to IL-1” and “response to decreased oxygen level” were enriched (Fig. 2J). These terms were akin to those of the PC2 genes, encompassing genes with similar expression patterns in vivo and the AO-EMO. The predicted upstream transcription factors involved in this regulation were *NFKB1* and *RELA* (fig. S2E). In epithelial cells of in vivo endometrial tissue, the expressions of p50 and p65 proteins encoded by these genes reach their peak during the mid-secretory phase (*37*, *38*). These results suggest that the collagen-based culture transiently creates an inflamed environment. This culture also induces gene expression patterns resembling those observed during endometrial tissue repair under hypoxic conditions (*39*). Building on these findings, we analyzed the impact of hypoxic conditions on EMO cultured in a collagen-based environment. When cultured under hypoxia, AO-EMO aggregation was enhanced, and the diameter of AO-EMO structures was reduced by day 7 of culture (fig. S2F). Comparative gene expression analysis revealed that GO terms related to inflammation, such as “response to lipopolysaccharide,” which were up-regulated in collagen-based cultures compared to Matrigel cultures, were also enriched in the gene sets up-regulated under hypoxia (fig. S2G). These observations suggest that genes associated with the hypoxic response may play a role in the formation of AO-EMO.

### Integrating stromal cells and endothelial network into AO-EMO

**Fig. 3.**
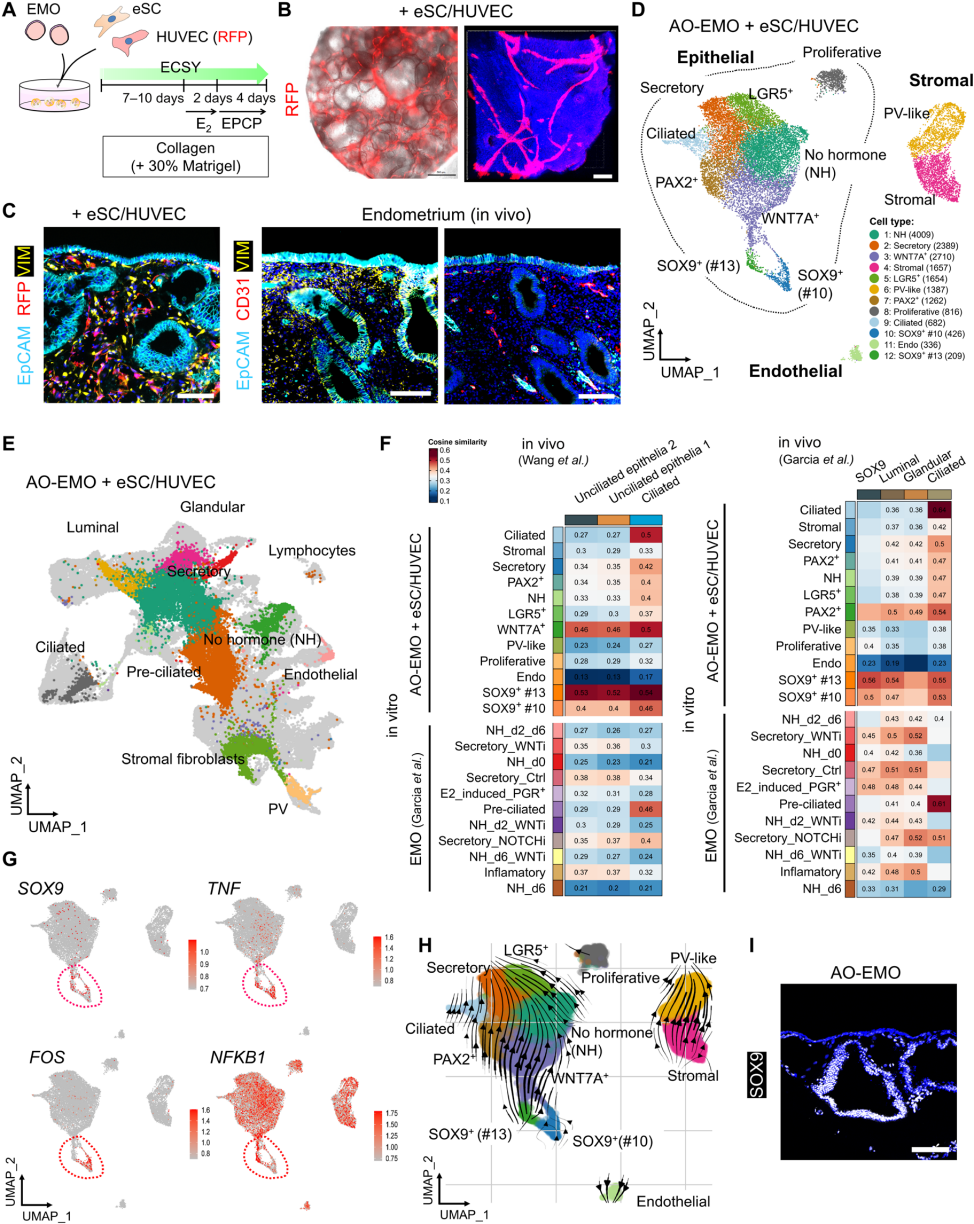
Integration of stromal cells and endothelial network into AO-EMO. (**A**) Schematic of the culture procedure. (**B**) Bright-field and fluorescence image of AO-EMO, including RFP-HUVEC. Scale bar, 500 μm (left). Representative image of maximum intensity projections of whole-mount stained AO-EMO including RFP-HUVEC; nuclei are stained with Hoechst (blue). Scale bar, 100 μm. (**C**) Fluorescence images of AO-EMO including eSC/RFP-HUVEC (left) and human endometrial tissues (right) stained for EpCAM, CD31, and VIM; nuclei are stained with Hoechst (blue). Scale bars, 100 μm. (**D**) UMAP plot of AO-EMO + eSC/HUVEC showing cell-type clusters. (**E**) UMAP plot representing the integrated data from AO-EMO + eSC/HUVEC, EMO (*30*), and in vivo endometrium preprocessed atlases (*30*, *35*). The light-gray–hued data points signify cells used in the multi-data fusion. (**F**) Heatmap illustrating the similarity between in vivo data sets (*x* axis) and integrated organoids (*y* axis), with color intensity representing the cosine similarity. (**G**) Feature plots showing expression levels and distribution of *SOX9*, *TNF*, *FOS*, and *NFKB1*. The red dashed line encircles SOX9^+^ cell populations. (**H**) Inferred directions from RNA velocity of AO-EMO + eSC/HUVEC embedded on UMAP coordinates. (**I**) Fluorescence image of AO-EMO stained for SOX9; nuclei are stained with Hoechst (blue). Scale bar, 100 μm.

The endometrial tissue comprises epithelial, stromal, and vascular endothelial cells, as well as immune cells, primarily uNK cells. This study introduces human eSC and human umbilical vein endothelial cells (HUVEC) to the AO-EMO to model these key cellular components other than immune cells. eSC, characterized by specific cell surface antigen expression and hormone-induced decidualization (fig. S3, A and B), were isolated from human endometrium or decidua and combined with EMO in collagen-based scaffolds (fig. S3C). The presence of eSC resulted in the protrusion of the EMO and an increase in contraction over time (fig. S3, D to F). Despite intermingling with eSC, epithelial cells emerged on the gel surface while exposing the apical surface, which exhibited ac. α-tubulin^+^ cilia, outwardly (fig. S3, G and H). Next, we cocultured HUVEC constitutively expressing red fluorescent protein (RFP) with EMO in collagen-based gels. Over time, HUVEC connected with each other and formed an endothelial network (fig. S3I). We confirmed the existence of a luminal structure with a cavity inside the organoids (fig. S3J). Furthermore, cleaved caspase-3 staining revealed that its coexistence with HUVEC suppressed apoptosis of epithelial and stromal cells inside the gel after a long (day 18) culture (fig. S3, K and L). Last, by mixing EMO, eSC, and HUVEC in a collagen-based gel, we generated a structure termed “AO-EMO + eSC/HUVEC” (Fig. 3, A to C).

We conducted a single-cell transcriptomic analysis to evaluate AO-EMO + eSC/HUVEC. We used single-nucleus RNA sequencing (snRNA-seq) following nuclear extraction and 4′,6-diamidino-2-phenylindole (DAPI)^+^ sorting (fig. S4A). On the basis of gene expression (fig. S4, B and C), we initially identified 14 distinct cell populations. Subsequently, these were categorized into 12 cell clusters, including various epithelial cell types (proliferative, secretory, ciliated, PAX2^+^, LGR5^+^, WNT7A^+^, no hormone, SOX9^+^-#10, and SOX9^+^-#13), stromal cells (stromal and perivascular-like), and endothelial cells (Fig. 3D). An integrated analysis of in vivo data (*30*, *35*) showed overlapping populations of these cell types (Fig. 3E). Comparing the proportions of each cell population in the organoids with in vivo data, we found that both AO-EMO and EMO had a lower proportion of luminal epithelial cell populations (fig. S4, D and E). Integrative analysis of AO-EMO and EMO unveiled a distinct population of SOX9^+^ cells within epithelial cell clusters in AO-EMO. In contrast, the proportion of estrogen-induced PGR^+^ cells and ciliated cells was lower in AO-EMO compared to that in EMO, suggesting that these cell populations were less likely to be induced in collagen-based cultures (fig. S4F). Cosine similarity metrics indicated that the SOX9^+^-#10 and SOX9^+^-#13 cell populations within AO-EMO displayed a considerable correlation with in vivo epithelial cells (Fig. 3F). Notably, the SOX9^+^-#13 population exhibited an especially robust correlation, underscoring its closer resemblance to the in vivo condition. Although the proportion of differentiated ciliated cells within AO-EMO was relatively small, these cells demonstrated a high correlation with in vivo ciliated cells, thus illustrating their similar characteristics. In SOX9^+^ epithelial cell populations, an enrichment of GO terms related to “unfolded protein binding” and “chaperone activity” was observed (fig. S4G). This cell population exhibited elevated expression of inflammation-related genes such as *TNF*, *FOS*, and *NFKB1* (Fig. 3G). This finding was consistent with the up-regulation of these genes in collagen-based gels identified by the bulk RNA-seq analysis, suggesting that collagen-based gels promote the emergence of these cell populations. RNA velocity analysis revealed the direction of differentiation within AO-EMO + eSC/HUVEC, indicating that the #13 cell population was particularly undifferentiated in main epithelial cell types (Fig. 3H). Furthermore, we analyzed the localization of SOX9^+^ cells within AO-EMO and found that they were predominantly located in the interior, with some cells on the surface or connected to surface cells (Fig. 3I). We also examined the effect of hormone supplementation. In the presence of hormones, terms related to epithelial cell migration were enriched, while genes with reduced expression in the presence of hormones were associated with cilia-related terms (fig. S4H). Furthermore, we observed an up-regulation of estrogen-responsive genes (*PGR* and *PCNA*) and secretory phase-related genes (*IL15*, *FOXO1*, *S100A4*, and *LMCD1*) (*16*) in the stromal cell populations upon hormone treatment (fig. S4I). Through an analysis of the AO-EMO snRNA-seq dataset, several potential interactions were observed between epithelial cells and both endothelial and stromal cells. Epithelial cells were noted to produce ligands such as vascular endothelial growth factor A (VEGFA), semaphorin 3C (SEMA3C), and platelet-derived growth factor C (PDGFC), which might target receptors including fms-like tyrosine kinase 1 (FLT1), platelet-derived growth factor receptor alpha (PDGFRA), neuropilin-1 (NRP1), plexin D1 (PLXND1), and EGF receptor (EGFR) in endothelial and stromal cells (fig. S5, A and B).

These results suggest that the inflammatory environment facilitated by collagen-based gels not only stimulates the formation of AO-EMO but also generates populations of undifferentiated SOX9^+^ epithelial cells that exhibit similarities in gene expression to in vivo epithelial cells. The localization of these cell populations also implies a potential involvement in forming AO structures. As a result, we have successfully established an in vitro model that mimics the luminal epithelial surface exposure, interstitial eSC, and endothelial network inherent to the in vivo endometrial structure. This model accurately represents the composition and spatial relationships of cells within the endometrium.

### Feto-maternal assembloids mimic human embryo implantation

**Fig. 4.**
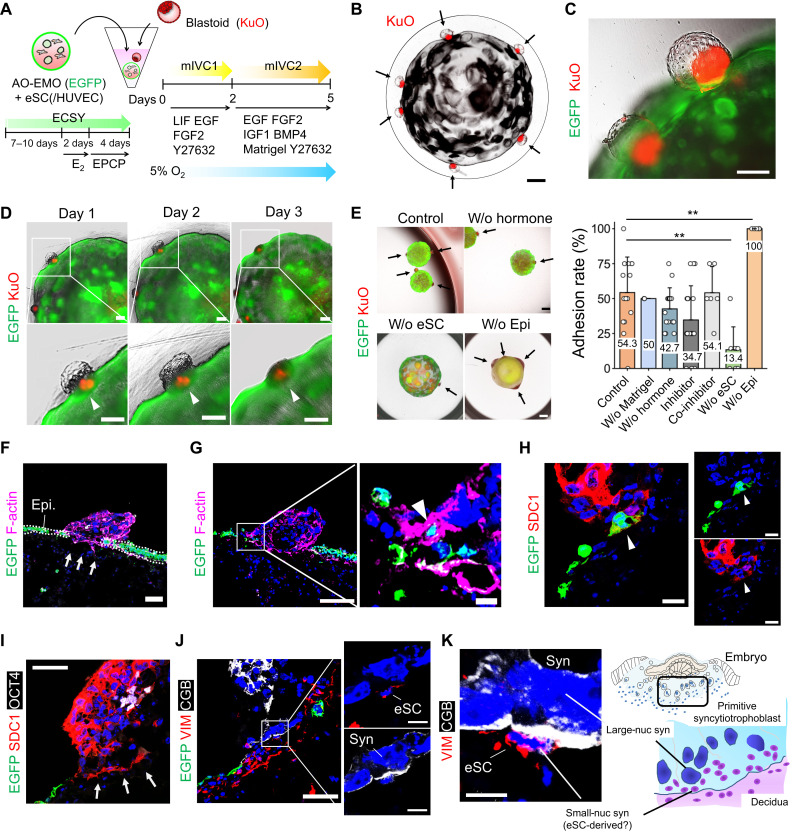
Feto-maternal assembloids mimic human embryo implantation. (**A**) Schematic of the coculture procedure. (**B**) Bright-field and fluorescence image of human blastoids (KuO^+^) and AO-EMO. Black arrows indicate blastoids. Scale bar, 500 μm. (**C**) Bright-field and fluorescence image of human blastoids (KuO^+^) and AO-EMO (Epi: EGFP^+^). Scale bar, 200 μm. (**D**) Combined bright-field and fluorescence images of human blastoids (KuO^+^) and AO-EMO (Epi: EGFP^+^) on days 1, 2, and 3 of coculture. Arrowheads indicate blastoids. Scale bars, 200 μm. (**E**) Bright-field and fluorescence images of blastoids (KuO) adhered to AO-EMO in various conditions (left). EGFP signals represent epithelial cells in all conditions except “w/o epi,” where they indicate eSC. Arrows point to attached blastoids. Scale bars, 500 μm. Quantification of attached blastoids (right). Data represent means ± SD; **P < 0.01; *n* = 15 (AO-EMO + eSC and w/o hormone), 3 (w/o Matrigel), 18 (inhibitor), 6 (co-inhibitor), 8 (w/o eSC), and 6 (w/o epi) independent coculture experiments. (**F** to **J**) Fluorescence images of feto-maternal assembloids (Epi: EGFP^+^); nuclei stained with Hoechst (blue). (F and G) Stained for F-actin. Scale bars, 50 μm and 10 μm (magnified). (H) Stained for SDC1. Arrowheads indicate SDC1^+^/EGFP^+^ cells. Scale bars, 20 μm. (I) Stained for SDC1 and OCT4. Scale bar, 50 μm. (J) Stained for VIM and CGB. Scale bars, 50 μm and 10 μm (magnified images). (**K**) Representative image of maximum projection of feto-maternal assembloid (Epi: EGFP^+^) stained for VIM and CGB; nuclei stained with Hoechst (blue) (left). Scale bar, 10 μm. Schematic representation of human embryo implantation and trophoblast invasion in decidua, highlighting the interface between primitive syncytiotrophoblasts and eSC (right).

After generating AO-EMO with exposed apical surfaces, we investigated their interaction with embryo surrogates. To track endometrial epithelial cells, we established EMO that constitutively express an enhanced green fluorescent protein (EGFP) via lentivirus-mediated gene transfer and cell sorting (fig. S6, A to C). We also generated Kusabira Orange (KuO)^+^–primed human ESC using lentivirus-mediated gene transfer and converted them into naïve state (fig. S6D). Subsequently, we introduced blastoids from KuO^+^ naïve ESC (*20*–*22*). While KuO expression was higher in the ICM-like cells, it was also detected in the TE-like cells, making it feasible to track cells derived from ESC in subsequent experiments (fig. S6E). Furthermore, we verified the quality of the blastoids by assessing induction efficiency, diameters, and the proportion of the three different cell lineages present and confirming the expression of nuclear receptor subfamily 2 group F member 2 (NR2F2), a marker for polar TE, on the ICM-like side of TE (fig. S6, F to H). We cocultured AO-EMO with eSC (with or without HUVEC) and blastoids in a floating culture. Ball-shaped endometrial models were transferred to a narrow well at the bottom, followed by the addition of blastoids. We adopted the in vitro culture (IVC)–based medium, specifically the modified IVC (mIVC) system reported by Xiang *et al.* (*40*–*42*). In addition, we included EGF and fibroblast growth factor (FGF) to promote AO-EMO + eSC/HUVEC growth, as well as leukemia inhibitory factor (LIF), which is reportedly involved in implantation (*43*). From day 2, we exogenously added insulin-like growth factor 1 (IGF-1), which promotes ICM proliferation (*44*), and bone morphogenetic protein 4 (BMP4), which is crucial for developing trophoblast lineage (*45*). We also included 10% Matrigel, similar to the report by Xiang *et al.* (*40*) (Fig. 4A). Using this culture system, blastoids were counter-located from the ICM side to the AO-EMO (Fig. 4, B and C). In continued cultures, the blastoids flattened as if they were entering the interior of the endometrial model (Fig. 4D). Blastoids that adhered to the endometrial model maintained their attachment even after being isolated and fixed (fig. S6I), and we termed this coculture product “feto-maternal assembloid.” We next quantified the adhesion rates of blastoids under various culture conditions (fig. S6J). Adhesion rates remained unchanged in the mIVC2 medium without Matrigel (w/o Matrigel). Without hormone pretreatment of the endometrial model (w/o hormone) or with the addition of estrogen and PGR inhibitors ICI 182,780 (1 μM) and Mifepristone (RU486) (10 μM), the adhesion rates showed a decrease to 42.7 and 34.7%, respectively, which were not statistically significant. Adhesion rates with inhibitors applied during coculture only (co-inhibitor) were similar to the control, at 54.1%. Notably, the absence of eSC led to a significant reduction in the adhesion rate (13.4%), whereas in the presence of eSC alone, without epithelial cells (w/o Epi), the adhesion was 100% (Fig. 4E). To investigate the pattern of blastoid adhesion, we performed staining with the ICM marker octamer-binding transcription factor (OCT4), confirming that blastoids adhered from the polar side in the presence of an endometrial model with eSC. Conversely, nearly half adhered from the mural side in the absence of epithelial cells (fig. S6K). These results suggest that the endometrial epithelium primarily acts as a barrier and blastoids that can adhere to it do so from the polar side.

Next, we conducted a detailed investigation of the feto-maternal assembloid. The endometrial epithelial cell layer was disrupted on the adherent surface of the blastoids (Fig. 4F and fig. S6L). In addition, EGFP^+^ endometrial epithelial cells were incorporated into blastoid structures, and an overlap between Syndecan-1 (SDC1)^+^ syncytial cells and EGFP^+^ endometrial epithelial cells was observed (Fig. 4, G and H). Whole-mount staining confirmed that the adhered blastoids contained SDC1^+^ syncytial cells appearing around the OCT4^+^ cells with an inner cavity and overlap with EGFP^+^ endometrial epithelial cells (fig. S6, M and N). Immediately below the structure of the OCT4^+^ ICM-like cells, SDC1^+^ syncytial cells infiltrated the interior of the endometrial model (Fig. 4I). Furthermore, the β-human chorionic gonadotropin (CGB)^+^ syncytial cells formed large nuclei and were in contact with the stromal cell marker vimentin (VIM)^+^ cells below them (Fig. 4J and fig. S6O). Such infiltration of the syncytial cells containing large nuclei is similar to the primitive syncytiotrophoblast structure observed during embryo implantation at the feto-maternal interface (Fig. 4K) (*46*, *47*).

These results indicated that our 3D coculture system, the feto-maternal assembloids, mimicked events at the interface between the embryo and the endometrium. The findings also suggest that blastoid-derived syncytium disrupts the endometrial epithelium and interacts with eSC during the invasion.

### Invading syncytium fuses with eSC

**Fig. 5.**
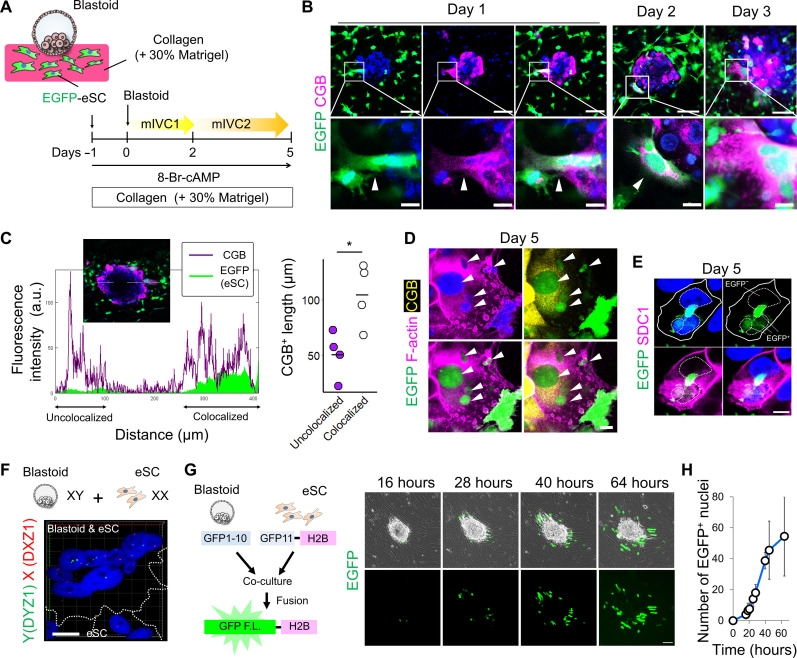
Interaction of the invading syncytium with eSC. (**A**) Schematic representation of the culture procedure. (**B**) Whole-mount imaging of blastoids cocultured with eSC (EGFP^+^) in a collagen-based gel on days 1, 2, and 3 stained for CGB; nuclei stained with Hoechst (blue). Scale bars, 100 μm and 20 μm (magnified). (**C**) Colocalization analysis of CGB^+^ cells and EGFP^+^ eSC (left). The *y* axis indicates fluorescence intensity, and the *x* axis shows distance. Quantification of CGB^+^ cell length colocalized with eSC or not (right); *n* = 4 independent experiments. *P < 0.05. a.u., arbitrary units. (**D** and **E**) Whole-mount imaging of blastoids cocultured with eSC (EGFP^+^) on day 5; nuclei stained with Hoechst (blue). Scale bar, 20 μm. (D) Stained for CGB and F-actin. Arrowheads indicate multiple EGFP^+^ nuclei in the same cell. (E) Stained for SDC1. Solid lines indicate cell boundaries; dashed lines mark nuclei. (**F**) X (DXZ1) and Y (DYZ1) chromosome detection of multinucleated cells in blastoid (SEES3-derived) cocultured with eSC. Maximum projection of fluorescence image; nuclei stained with Hoechst (blue). Scale bar, 20 μm. The dashed lines delineate the outlines of the cells. (**G**) Schematic of experimental design (left). Phase contrast and fluorescence images of attached blastoid expressing GFP1-10 cocultured with eSC expressing GFP11-tagged histone H2B at 16, 28, 40, and 64 hours after coculture (right). Scale bar, 100 μm. (**H**) Quantification of EGFP^+^ nuclei during coculture; *n* = 4 independent coculture experiments.

The feto-maternal assembloids suggest the contact between syncytial cells and eSC. To investigate the interaction between these two cell types further, we performed a simple 3D coculture. Before coculturing with blastoids, eSC expressing EGFP were embedded in a collagen-based gel. Subsequently, the blastoids were placed on the gel surface and cocultured in the mIVC1/mIVC2 medium. For the decidualization of eSC, 8-Br-cAMP was added to these media, which contained E_2_ and progesterone (Fig. 5A). This coculture resulted in the emergence of chorionic gonadotropin subunit beta (CGB)^+^ syncytial cells at the outer edge of the blastoids, which extended toward the surrounding eSC, with fusion of EGFP and CGB signals. On day 2 of the coculture, CGB signals were detected in the EGFP^+^ eSC surrounding the blastoids, indicating that the cytoplasmic components of the syncytial and stromal cells were mixed. After 3 days of coculture, the signal of CGB fused with EGFP expanded more radially (Fig. 5B and fig. S7A). Colocalization analysis showed that CGB^+^ syncytial cells were elongated when colocalized with EGFP^+^ eSC (Fig. 5C). By day 5, multinucleated cells, characterized by EGFP^+^ nuclei and CGB^+^ cytoplasm, were prominent at the blastoid-eSC interface (Fig. 5D and fig. S7B). In addition, several EGFP signals were detected inside the cells expressing SDC1 on the cell membrane (fig. S7C). Furthermore, within the cell regions enclosed by the SDC1-detectable cell membrane, both an EGFP^−^ nucleus derived from blastoids and EGFP^+^ nuclei from eSC were observed (Fig. 5E). These results indicate that cellular fusion occurs between syncytial cells and eSC. We confirmed that this fusion was not dependent on the addition of 8-Br-cAMP, an agonist of cAMP signaling triggering trophoblast cell fusion (fig. S7D) (*48*). Detailed analysis by confocal microscopy showed that ~70% of CGB^+^ cells merged with GFP^+^ eSC, whereas OCT4^+^ cells showed little or no merged signals, suggesting that syncytial cells among blastoid-derived cells fuse with eSC (fig. S7E). We also found that a substantial proportion of the fused cells, defined as EGFP^+^/CGB^+^, lacked the signal for VIM, a conventional marker for stromal cells. This absence of VIM in fully fused cells implies a comprehensive loss of their stromal cell identity, indicating a potential shift in their cellular role or phenotype (fig. S7, F and G). The 2D coculture also demonstrated that when blastoids were seeded onto eSC, the area occupied by the blastoids increased, particularly that of CGB^+^ syncytial cells (fig. S8, A to C). A detailed analysis of the interface between the blastoids and eSC showed that the CGB^+^ syncytial cells surrounded the EGFP^+^ nuclei of eSC (fig. S8D). Moreover, we converted a male ESC line (SEES3), which has the XY pair of sex chromosomes, into the naïve state and then induced blastoids for coculture with eSC (fig. S8E). By the fourth day of coculture, using DNA–fluorescence in situ hybridization (FISH) and F-actin cytoplasmic staining, we confirmed the presence of multinucleated cells at the boundary between the blastoids and eSC, with the blastoid-derived nuclei bearing Y chromosomes and the eSC-derived nuclei containing only X chromosomes (Fig. 5F and fig. S8F). Furthermore, we used a split–green fluorescent protein (GFP) system (*49*). Briefly, GFP1-10 was introduced into primed eSC, and blastoids were induced after converting to a naïve state (fig. S8G). These were then cocultured with eSC, into which GFP11-tagged histone H2B had been introduced. As the culture continued, EGFP^+^ cells emerged from the periphery of the attached blastoids. Over time, the number of these positive cells increased in a radial pattern (Fig. 5, G and H). Because GFP fluorescence could not be detected unless cells from the blastoid and the eSC fused, this confirmed that these cells were indeed fusing. These results suggest a potential mechanism for the invasion of the syncytium, in which eSC are fused during implantation.

### Culture and analysis of human blastocysts for investigating interaction with endometrial cells

**Fig. 6.**
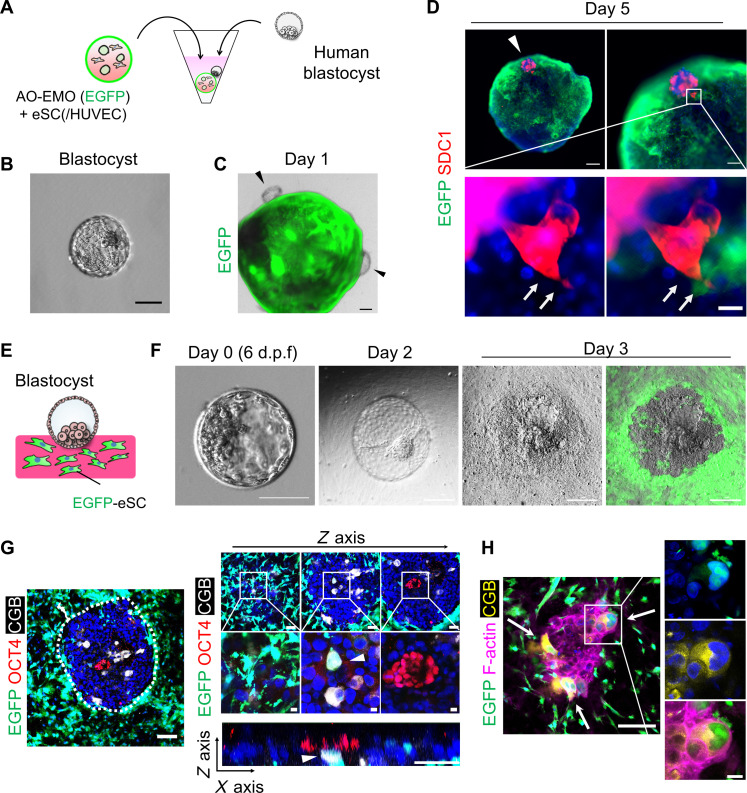
Culture and analysis of human blastocysts for investigating interaction with endometrial cells. (**A**) Schematic representation of the culture procedure. (**B**) Bright-field image of a human blastocyst. Scale bar, 100 μm. (**C**) Combined bright-field and fluorescence image of human blastocyst in coculture with AO-EMO (Epi: EGFP^+^) on day 1. The arrowheads point to blastocysts. Scale bar, 200 μm. (**D**) Whole-mount fluorescence imaging of the blastocyst attached to AO-EMO + eSC (Epi: EGFP^+^) stained for SDC1; nuclei stained with Hoechst (blue). Scale bars, 200 μm (top left), 100 μm (top right), and 20 μm (bottom right). The arrowhead indicates the attached blastocyst; the arrows indicate the infiltrating syncytial cells. (**E**) Schematic representation of the culture procedure. (**F**) Bright-field and fluorescence images of human blastocysts and eSC (EGFP^+^). Scale bars, 100 μm. d.p.f., days postfertilization. (**G**) Whole-mount imaging of human blastocyst cocultured with eSC (EGFP^+^) in collagen-based gel on day 3 stained for OCT4 and CGB; nuclei stained with Hoechst (blue). The bottom right panel shows a cross-sectional view along the *x* and *z* axes. Arrowheads indicate EGFP^+^/CGB^+^ cells. Scale bars, 100 μm (left and top right), 10 μm (magnified), and 50 μm (*xz* plane). (**H**) Whole-mount imaging of human blastocyst cocultured with eSC (EGFP^+^) in a collagen-based gel on day 3 stained for CGB and F-actin; nuclei stained with Hoechst (blue). Arrows point to EGFP^+^/CGB^+^ cells. Scale bars, 100 μm and 20 μm (magnified).

To confirm the phenomena observed in blastoids, we applied the coculture system in this study to human blastocysts. Initially, we cocultured human blastocysts with the AO-EMO model in floating culture (Fig. 6, A and B). Human blastocysts nestled against the endometrial model, similarly to blastoids, and, although their adhesion rates were lower compared to those of blastoids (fig. S9A), adhesion was confirmed on the fifth day of coculture (Fig. 6C). In addition, SDC1^+^ syncytial cells emerging from the blastocysts appeared to infiltrate between the EGFP^+^ endometrial epithelial cells (Fig. 6D).

Next, we investigated the syncytial and stromal cell fusion observed in blastoid experiments using human blastocysts (Fig. 6E). Upon placement on a collagen-based gel containing EGFP^+^ eSC, blastocysts adhered to the gel surface from the ICM side within 2 days of coculture and exhibited a flattened morphology by day 3 (Fig. 6F and fig. S9, B and C). Differentiation of CGB^+^ cells was observed at the interface between OCT4^+^ cells immediately subjacent to the blastocyst and eSC at the blastocyst periphery, leading to fusion with EGFP^+^ cells (Fig. 6G and fig. S9, D and E). Even when cell boundaries were detected with F-actin staining, signals of both EGFP and CGB coexisted within the same cell region (Fig. 6H). Thus, by using human blastocysts, we successfully replicated phenomena exhibited by human embryonic cells, such as adhesion and invasion to the endometrial epithelium, as well as fusion with eSC.

## DISCUSSION

Organoids like EMO offer better tissue modeling than monolayer cultures (*23*, *24*), but accessing their apical surface is difficult. In this study, we developed a simple methodology for fabricating AO organoids using a collagen-based substrate, enabling endometrial epithelial cells to migrate to the surface and expose the apical surface. AO-EMO display spatial heterogeneity, with higher surface epithelial markers externally and glandular epithelial markers internally. Furthermore, the AO-EMO allow secretions from the internal gland-like epithelial cells to diffuse directly to the exterior of the organoid. We also successfully incorporated dense eSC and endothelial networks into AO-EMO. Epithelial cells of the AO-EMO express SOX9, resembling their in vivo counterparts. Notably, *SOX9* can be up-regulated by nuclear factor κB (*50*). In line with this, the SOX9^+^ epithelial cell population (#10 and #13) exhibited increased expression of inflammation-related genes, such as *TNF* and *FOS*. The inflammatory environment may contribute to elevated SOX9 expression. Although luminal epithelium and other cell types remain immature in vitro, modulating signaling pathways and improving culture conditions could potentially generate more mature organoids. AO-EMO addresses limitations of conventional organoids (*4*), such as apical accessibility, stromal cell scarcity, and vascularization challenges, making them valuable for studying embryo implantation and endometrial biology.

To study human embryo implantation, various in vitro models have been developed (*51*). One notable approach involves the creation of 3D endometrial constructs by embedding eSC in collagen and then overlaying them with epithelial cells (*26*). These constructs have been used in coculture systems with human embryos to investigate the impact of contraceptives on embryo adhesion (*52*–*54*). Implantation involves a dynamic interaction between two distinct tissues: the developing embryo and the maternal endometrium. To explore these intricate tissue interactions, assembloid-based analyses offer an alternative yet effective research avenue. For example, the migration of interneurons and synaptic connections in the brain are reproduced by assembloid works (*55*, *56*). A coculture system incorporating floating cultures might facilitate autonomous interactions, triggering an internalized, self-directed developmental program. In this study, we used a 3D floating coculture system combining blastoids and AO-EMO to explore feto-maternal interactions. Although the presence or absence of hormones in the endometrial model did not significantly affect the adhesion of blastoids, we found that all blastoids adhered in the absence of endometrial epithelium in our endometrial model. These findings are consistent with the observation that human embryos can adhere to and invade a variety of tissues, with the endometrium serving as the sole barrier (*57*). Intriguingly, blastoids exhibited polar adhesion when in contact with an AO-EMO + eSC. In contrast, in the absence of epithelium, the directionality of their adhesion became disordered, highlighting the essential role of the endometrial epithelium in regulating proper embryonic adhesion for successful development. Furthermore, we observed a marked decrease in adhesion rates when eSC were not included in the endometrial model. During the implantation window, there is such a high density of stromal cells beneath the epithelial cells in the endometrium that it is referred to as “edema.” In traditional flat models, including stromal cells at a density similar to that under in vivo conditions was difficult due to gel contraction. Because of its self-aggregating nature, the sphere-like model with AO-EMO containing eSC can maintain an internal environment with a high density of stromal cells. Molecules derived from stromal cells, as visualized in our ligand-receptor analysis, may play a role in reducing the barrier function of the endometrial epithelium. Consequently, the implementation of a 3D floating culture in our feto-maternal assembloids, designed to foster autonomous cellular interactions, underscores the pivotal and dynamic contribution of endometrial epithelial and stromal cells in the regulation of implantation processes.

Our observations of integrating EGFP^+^ endometrial epithelial cells into the syncytium using these assembloids provided insights not discernible in previous 2D implantation models (*15*, *22*). It has been demonstrated that the presence of endometrial epithelium promotes the differentiation of syncytiotrophoblast cells (*15*). In addition, single-cell analysis indicated that TE cells on the side that adheres to the endometrial epithelium, namely, polar TE in humans and mural TE in mice, display up-regulated expression of syncytial cell–related genes (*58*). Integrating our findings with prior research, we suggest that syncytial differentiation at the adhesion surface of the embryo and the incorporation of endometrial epithelium play crucial roles in breaching the epithelial barrier and promoting endometrial invasion. As a result, the degree of syncytial differentiation may be a determining factor in both the TE attachment site and implantation rate. Future studies investigating the impact of manipulating syncytial differentiation in blastoids or blastocysts on adhesion rates could reveal key factors for successful implantation. In addition, we applied the AO-EMO coculture system to human blastocysts, confirming adhesion and syncytial cell invasion. However, a notable limitation of our study is the lower adhesion rate in blastocysts compared to blastoids, indicating the need for a greater number of blastocysts for more detailed analysis, which must be considered alongside ethical implications. Another limitation is that feto-maternal assembloids could not be cultured until the blastoids were fully embedded in the endometrial models. Consequently, structures indicative of embryonic-lineage progression, such as the yolk sac, extraembryonic mesoderm, or primitive streak, were not observed. In addition, the optimality of our culture conditions or the essentiality of the added factors could not be validated. Therefore, further research is essential to assess the developmental potential and limitations of blastoids more comprehensively.

In this study, we successfully confirmed the fusion between syncytial cells and eSC using human blastoid and blastocysts through a multifaceted approach. In human embryo tissue sections implanted in the uterus, stromal cells have been observed beneath the syncytium (*47*). In addition, a small nuclear syncytium is present at the interface between the primitive syncytium and the endometrial stroma (*46*). The exact nature of this small nuclear syncytium has yet to be elucidated (*59*). Our findings support the hypothesis that it may arise from the fusion of fetal and maternal cells, potentially incorporating stromal cells during the invasion process. We postulate that the efficient integration of dense stromal cells beneath the trophoblast during embryo implantation and invasion into the maternal uterus could facilitate this process. Our data revealed that the fused cells lose their stromal cell identity, as evidenced by the absence of the stromal cell marker VIM. VIM can still be detected when the syncytial and stromal cells come into contact or at the beginning of fusion, but VIM expression may not be maintained when they are fully fused. Consequently, double-staining techniques using specific stromal and syncytial cell markers may not detect this fusion, given that the fused cells no longer express stromal cell marker proteins. Nonetheless, our study demonstrates that using EGFP-expressing stromal cells serves as an effective method for detecting such fusion events. In the human placenta, mononuclear cytotrophoblasts undergo a tightly regulated fusion process to form multinucleated syncytiotrophoblasts, orchestrated spatiotemporally by key proteins such as cadherin and syncytin, which are endogenous retroviruses (*48*). Notably, human embryo implantation is characterized by extensive uterine infiltration (*60*), with the degree of syncytial and maternal fusion potentially determining the extent of infiltration. Investigating the occurrence of these cells’ fusion in vivo will be crucial in future studies.

**Fig. 7.**
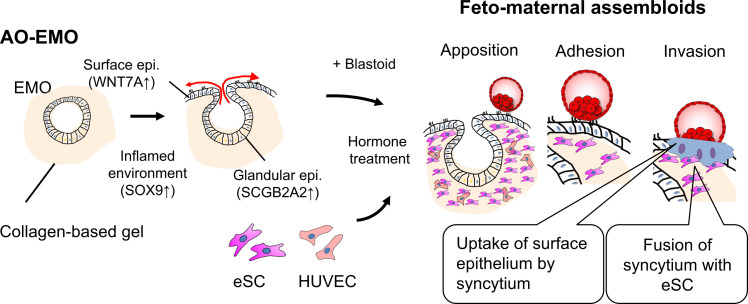
Schematic representation of the endometrial and implantation models developed in this study.

In summary, we have developed a 3D coculture system, feto-maternal assembloids, using the blastoids and AO-EMO model that closely resembles the endometrial tissue. This system allows visualization of the embryo-endometrium boundary and suggests that fetal and maternal cell fusion may occur during early implantation (Fig. 7). As this system comprises a diverse range of fetal and maternal cell types, it will allow the manipulation of the molecules in both fetal and maternal cells through transfection and genome editing, thereby facilitating the examination of the molecular mechanisms involved in implantation. Advancements in assisted reproductive technology have not fully addressed global infertility challenges. As such, the refinement of coculture systems and the development of infertility models that inhibit implantation could present a viable approach to addressing implantation-related issues. This, in turn, could potentially enhance the success rate of IVF procedures and provide solutions for recurrent implantation failure.

## MATERIALS AND METHODS

### Human samples

Human endometrium and decidua (6 to 9 weeks of gestation) samples were obtained from donors with written informed consent. All experiments involving human participants were approved by the Ethics Committee of Tohoku University School of Medicine (research license 2022-1-238). Human blastocysts were obtained with signed informed consent from the donors and approved by the Ethics Committee of Tohoku University School of Medicine (research license 2022-1-1012).

### Cell lines

The primed human ESC lines (SEES1 and SEES3) were provided by H. Akutsu and A. Umezawa (National Center for Child Health and Development, Tokyo, Japan). All experimental protocols and procedures were approved by the Ethics Committee of the Tohoku University Graduate School of Medicine (2022-1-704). HUVEC and RFP-HUVEC were purchased from Takara Bio (Kyoto, Japan) and Angioproteomie (Boston, MA), respectively. 293T cells were purchased from Takara Bio.

### Generation of human EMO and isolation of human eSC

Human EMO were generated on the basis of previously described methods (*23*, *24*). Endometrial or decidual tissue fractions were minced and incubated for 1 hour in an enzyme solution [collagenase V, Sigma-Aldrich, C-9263; dispase II (0.5 mg/ml), Sigma-Aldrich, D4693; and 10% fetal bovine serum (FBS; 1.5 U/ml) in RPMI 1640 medium] with shaking. Thereafter, culture medium [Dulbecco’s modified Eagle’s medium (DMEM)/F-12 supplemented with 10% FBS] was added to stop enzymatic reactions, and the cells were passed through a 100-μm filter.

To generate EMO, the filter was inverted, and the trapped cells were collected and washed with the medium. The cell pellets were mixed with 20 volumes of ice-cold Matrigel (Corning, NY, USA). The sample was added as 20-μl drops onto a pre-warmed 48-well plate. The samples were incubated at 37°C for more than 15 min, and 250 μl of medium was added. EMO were cultured as described previously (*23*, *24*), with minor modifications. The EMO medium comprised the following: advanced DMEM/F-12 (Life Technologies) supplemented with 1% ITS-X supplement (FUJIFILM Wako), 1% N-2 supplement (Life Technologies), 2% B27 supplement (Life Technologies), 1 mM nicotinamide (FUJIFILM Wako), 1 mM *N*-acetyl-l-cysteine (FUJIFILM Wako), 1× GlutaMAX supplement (Gibco), heparin (1 μg/ml; FUJIFILM Wako), 0.2 mM l-ascorbic acid (FUJIFILM Wako), 10 μM Y27632 (FUJIFILM Wako), 2 μM CHIR99021 (FUJIFILM Wako), 2 μM SB202190 (FUJIFILM Wako), 0.5 μM A83-01 (FUJIFILM Wako), EGF (50 ng/ml; FUJIFILM Wako), FGF2 (50 ng/ml; FUJIFILM Wako), FGF10 (50 ng/ml; PeproTech), Noggin (100 ng/ml; PeproTech), and 1% penicillin/streptomycin (Gibco). EMO were also established and maintained in the ECSY medium. The composition of the ECSY medium was as follows: DMEM/F-12 (Life Technologies) supplemented with 1% ITS-X supplement, 0.15% bovine serum albumin (BSA; FUJIFILM Wako), 1% Knockout Serum Replacement (KSR; Thermo Fisher Scientific), 200 mM l-ascorbic acid (FUJIFILM Wako), EGF (50 ng/ml), 2 μM CHIR99021, 2 μM SB202190, and 10 μM Y27632. EMO were maintained at 37°C in a 5% CO_2_ in air atmosphere.

The cells that passed through the 100-μm filter were refiltered using a 40-μm filter to isolate stromal cells. The cells obtained after the second filtration were seeded onto a T-75 flask (BD Falcon) and cultured with 10% FBS containing DMEM/F-12. After 18 hours, the medium was changed to remove the blood cells, debris, and epithelial cells.

### Generation of AO-EMO and inclusion of human eSC and HUVEC

Ice-cold DMEM/F-12 was added to the EMO maintained in Matrigel and thoroughly homogenized by repetitive pipetting (~20 to 50 times). The EMO fragments were centrifuged at 500*g* for 3 min, after which the supernatant was meticulously aspirated. Subsequently, a blend of 70% type I collagen (CellMatrix TypeI-A, 3 mg/ml; Nitta Gelatin) and 30% Matrigel was incorporated into a pellet of EMO fragments, and 20 μl of the resulting mixture was dispensed into a nontreated 48-well plate. The plates were then incubated at 37°C for 30 min. ECSY medium supplemented with FGF2 (50 ng/ml; FUJIFILM Wako) and VEGF (20 ng/ml; FUJIFILM Wako) was added and replaced every 2 or 3 days. AO-EMO were cultured at 37°C in a 5% CO_2_ in air atmosphere. To isolate the outer and inner cells of AO-EMO, the culture medium was aspirated, and phosphate-buffered saline (PBS) was added for washing. Subsequently, TrypLE Express was added to the culture dish, and plates were incubated at 37°C for 30 min. Following the incubation, TrypLE was removed, and AO-EMO were washed with PBS. The outer cells were detached by pipetting PBS onto the surface of the AO-EMO several times using a manual pipette.

Human eSC and HUVEC were added to pellets of EMO fragments to include these cells and a collagen-based gel to achieve a density of 2 × 10^7^ (human eSC) and 1 × 10^7^ (HUVEC) cells/ml. These mixed gels were cultured in a 48-well plate as described above.

### Immunofluorescence staining

Cells and organoids were fixed with 4% paraformaldehyde (PFA) and washed thrice with PBS. The cells and frozen sections were incubated with PBS containing 5% normal goat serum and 0.3% Triton X-100 for 1 hour at room temperature to block nonspecific reactions. They were incubated with primary antibodies (table S1) overnight at 4°C and stained with Alexa Fluor 488–, Alexa Fluor 555–, and Alexa Fluor 647–conjugated secondary antibodies (Cell Signaling Technology). The nuclei were stained with Hoechst 33342 (Molecular Probes); F-actin was stained with a red fluorescent dye (cytoskeleton) or iFluor647 (Cayman Chemical)–conjugated phalloidin. Stained samples were observed using a BZ-X810 (Keyence) or LSM780 (Carl Zeiss). The images were analyzed using a Fiji (*61*) and BZ-X800 analyzer (Keyence).

### Immunohistochemistry

Human endometrial tissues were fixed with 4% PFA for 2 days at 4°C, washed with PBS, treated with ethanol series, embedded in paraffin, and cut into 5-μm-thick sections. After deparaffinization and hydration, the sections were then treated with HistoVT One (Nacalai Tesque) at 90°C for 20 min for antigen retrieval. The slides were then cooled and washed with PBS. Subsequently, endogenous peroxidase activity was blocked using a 3% H_2_O_2_ solution for 20 min. After washing with PBS, nonspecific staining was blocked by incubating the slides in PBS containing 5% normal goat serum and 0.3% Triton X-100 for 1 hour. The slides were stained with primary antibodies overnight at 4°C. After washing slides three times with PBS, the antigen-antibody complexes were visualized using Histostar (MBL) and diaminobenzidene (DAB) substrate solution (MBL). The slides were counterstained with hematoxylin (FUJIFILM Wako), and images were captured using a BZ-X810 microscope (Keyence). The antibodies used in this study are shown in table S1.

### Quantitative real-time reverse transcriptase polymerase chain reaction

Total RNA was extracted from the cells using the RNeasy kit or QIAzol reagent (QIAGEN, Valencia, CA, USA). The PrimeScript II first Strand cDNA Synthesis Kit (Takara Bio) was used to synthesize cDNA. Quantitative reverse transcriptase polymerase chain reaction (PCR) was performed using the StepOnePlus Real-Time PCR System (Applied Biosystems, Foster City, CA, USA) and SYBR Premix Ex Taq II (Takara Bio) according to the manufacturer’s instructions. The amount of target mRNA was determined using the ΔΔCt (threshold cycle) method with glyceraldehyde-3-phosphate dehydrogenase (*GAPDH*) or beta-2-microglobulin (*B2M*) as the internal control. The primers used in this study are shown in data S1.

### Enzyme-linked immunosorbent assay

The passaging EMO were fragmented using a basal medium (DMEM/F-12) and then divided into equal volumes for culture in Matrigel or collagen-based gels. After 7 to 14 days of culture, E2 was added. Two or 3 days later, EPCP [10 nM β-estradiol (E2), 1 μM MPA, 50 μM 8-Br-cAMP, and prolactin (50 ng/ml)] was added, and the cells were cultured for 3 days. Afterward, the supernatant was collected, centrifuged at 500*g*, and the supernatant was stored at −80°C. The amount of secreted glycodelin was measured using a Human PP14 (PAEP) enzyme-linked immunosorbent assay kit (RayBiotech), according to the manufacturer’s protocol. Absorbance was measured using a FlexStation 3 (Molecular Devices).

### Bulk RNA-seq and analysis

Total RNA was extracted using the RNeasy Mini or Micro Kit (QIAGEN), and genomic DNA was removed by digestion with ribonuclease-free deoxyribonuclease I (QIAGEN). RNA libraries were prepared using the NEBNext UltraII Directional RNA Library Prep Kit (New England Biolabs). RNA integrity was assessed using a TapeStation 2200 (Agilent Technologies). The libraries were sequenced on the Illumina Hiseq2500 or NovaSeq 6000 platform (Illumina) with 150–base pair (bp) paired-end reads. The sequenced data were first trimmed for quality control using the TrimGalore software (v0.6.7). The reads were then aligned to the reference genome (UCSC hg38) using STAR (v2.7.10a) (*62*) with the RefSeq gene annotation. The expression levels (transcript per million) of the Refseq genes were calculated using RSEM (v1.3.1) (*63*). Read counts were used to identify differentially expressed genes using DESeq2 (*64*). The PCA was performed using the “prcomp” function in R. GO term, KEGG pathway, and GSEAs were performed using the “cluterProfiler” package (v4.8.0) in R (*65*). Gene enrichment analysis was performed using MetaScape (v3.5).

### snRNA-seq and analysis

AO-EMO + eSC/HUVEC were collected into a LoBind 1.5-ml tube and subjected to two PBS washes. Afterward, 1 ml of Nuclei EZ lysis buffer (Sigma-Aldrich) was added, and the AO-EMO were homogenized using a plastic pestle with 10 to 20 strokes. The solution was then transferred to a 15-ml tube (BD Falcon) using a wide-bore pipette, 2 ml of Nuclei EZ lysis buffer was added, and the mixture was incubated on ice for 5 min with gentle mixing using a pipette. The solution was passed through a 35-μm filter, and the resulting supernatant was obtained through centrifugation at 500*g* for 5 min at 4°C. Afterward, the pellet was gently resuspended in 1.5 ml of Nuclei EZ lysis buffer and incubated on ice for 5 min before being subjected to another centrifugation step, as described above. The final pellet was washed and resuspended in 1 ml of Nuclei Wash and Resuspension Buffer (Sigma-Aldrich) and passed through a 35-μm filter. Then, 1 μl of DAPI solution (10 μg/ml) was added, and the nuclei were visually inspected on a hemocytometer. A 10-μl aliquot of the suspension was added to a C-chip (VWR), and nuclei were counted using ImageJ software. DAPI^+^ nuclei were sorted using a FACSAria II cell sorter (BD Biosciences) and collected in low-cell adsorption tubes (STEMFULL, Sumitomo Bakelite). The sample was centrifuged at 500*g* for 5 min at 4°C, and the supernatant was meticulously removed. Subsequently, 1 ml of Nuclei Wash and Resuspension Buffer was added, and the sample was again centrifuged at 500*g* for 5 min at 4°C. The resulting supernatant was discarded, and 300 μl of Nuclei Wash and Resuspension Buffer were added to the sample. The nuclei were then quantified, and the sample was centrifuged at 500*g* for 5 min at 4°C. The nuclei concentration was adjusted to 1000 nuclei/μl, and 10,000 single nuclei were subsequently loaded onto a Chromium Controller (10x Genomics) for further analysis.

Single-cell libraries were prepared using the Chromium Single Cell 3′ Reagent Kits v3.1 for Dual Index (10x Genomics), according to the manufacturer’s instructions. Briefly, sorted nuclei in suspension were first prepared as gel beads in emulsion (GEMs) on a Chromium Next GEM Chip G (10x Genomics) using a Chromium Controller (10x Genomics). Barcoded RNA transcripts in each single cell were reverse-transcribed within GEM droplets. cDNA was purified using DynaBeads MyOne Silane beads (Invitrogen) and amplified for subsequent library construction. Sequencing libraries were prepared by fragmentation, end repair, ligation with adapters, and PCR amplification using the Library Construction Kit (10x Genomics). Nucleic acids were cleaned up after each step using SPRIselect beads (Beckman Coulter). The pooled libraries were sequenced using an Illumina NovaSeq 6000. All single-cell libraries were sequenced with a paired-end dual-index format (150 bp) according to the manufacturer’s instructions.

Raw scRNA-seq reads were mapped to genome sequences using the CellRanger pipeline (v7.1.0) (*66*). The human genome and annotation (GRCh38-2020-A-2.0.0) were obtained from 10x Genomics. We used Velocyto (v0.17.17) (*67*) to obtain spliced/unspliced expression matrices included in loom files. We merged the loom files with Loompy (v3.0.7) and converted the merged file into a Seurat object (v4.1.3) (*68*). Quality check preprocessing and data integration were performed using Seurat (v4.3.0) (*68*). We filtered out cells that express less than 1000 or more than 6000 genes, along with cells with a proportion of mitochondrial RNA larger than 1% and unique molecular identifier (UMI) counts inferior to 3200 from the downstream analysis. Cell cycling was also considered and, along with the mitochondrial content, was regressed out during the normalization process conducted using SCTransform (v0.3.5) (*69*). Dimension reduction was performed via uniform manifold approximation and projection (UMAP) (*70*) using the top 30 principal components from PCA. We tested multiple resolutions for Louvain graph–based clustering (0.1, 0.2, 0.3, 0.5, 0.6, 0.8, and 1) and chose 0.8 for further cluster identification. Known marker genes were obtained from reference [Genetic Algorithm (GA) reference], and other marker genes were calculated using the “FindMarkers” feature of Seurat with a log fold-change threshold of 0.25 and min.pct of 0.25. Only significant genes were used in further analysis (*P* adjusted < 0.05). GO term enrichment analysis was performed on the gene sets [*P* < 0.01, *q* < 0.01, false discovery rate (FDR) calculated with Benjamini-Hochberg] using ClusterProfiler (v4.6.0) (*71*). For cell clusters annotation, we also compared our annotated clusters to the cell atlases of the human EMO and human in vivo endometrium (*30*, *35*). Public datasets were downsized to similar number of cells maintaining the original cell-type proportion. The calculation of cosine similarity scores and UMAP plots were generated using the SCP package (v0.2.7). Trajectory analysis was performed with scVelo (v0.2.5) (*72*) using “dynamical model” mode, and the UMAP coordinates were imported from the Seurat object using the package SeuratDisk (v0.0.0.9020).

### Human ESC culture

Primed human ESC were maintained on Matrigel in StemSure hPSC medium (FUJIFILM Wako) supplemented with FGF2 (35 ng/ml; FUJIFILM Wako). After passage, 10 μM Y27632 was added to the medium for 24 hours. Naïve human ESC were generated from primed human ESC by culturing in 5i/L/A (*73*): Dissociated primed human ESC were seeded on mitomycin C–treated SNL (Cell Biolabs) feeder layer in DMEM/F-12 supplemented with 15% FBS, 5% KSR, 1 mM glutamine (Thermo Fisher Scientific), 1% nonessential amino acids (Thermo Fisher Scientific), 0.5% penicillin/streptomycin, 0.1 mM 2-mercaptoethanol, FGF2 (4 ng/ml), and 10 μMY27632. After 1 day, the medium was switched to N2B27 medium [1:1 DMEM/F-12 and Neurobasal (Thermo Fisher Scientific) medium supplemented with 1% N-2 supplement, 2% B27 supplement, 1% nonessential amino acids, 1 mM glutamine, 0.1 mM 2-mercaptoethanol, 0.5% penicillin/streptomycin, and BSA (50 μg/ml)] supplemented with 5i/L/A [LIF (20 ng/ml; FUJIFILM Wako), activin A (20 ng/ml; FUJIFILM Wako), 1 μM PD0325901 (FUJIFILM Wako), 1 μM IM-12 (FUJIFILM Wako), 0.5 μM SB590885 (Adipogen Life Sciences), 1 μM WH-4-023 (FUJIFILM Wako), and 10 μM Y27632]. Naïve human ESC were maintained on a mitomycin C–treated SNL or mouse embryonic fibroblast (MEF) feeder layer in 5i/L/A medium under 5% CO_2_ and 5% O_2_ conditions. Naïve human ESC were passaged every 5 to 7 days via single-cell dissociation using a 5-min treatment with Accutase (Innovative Cell Technologies).

### Lentivirus production and infection

*EGFP* was PCR-amplified from pEGFP-N1 (Clonetech) and cloned into the CS-CA-MCS plasmid (provided by H. Miyoshi, RIKEN BioResource Center, Ibaraki, Japan) using the In-Fusion HD Cloning Kit (Takara) to generate pCS-CA-EGFP (*74*). pCS-CA-EGFP was cotransfected with pCMV-VSV-G-RSV-Rev and pCAG-HIV-gp (provided by H. Miyoshi) into 293T cells (Takara Bio) using Lipofectamine LTX (Thermo Fisher Scientific) or CalFectin (Signagen). Forskolin (10 μM) was added after 24 hours of transfection. The supernatant was collected 3 days after transfection and concentrated using a Lenti-X Concentrator (Takara Bio). The primers used for cloning are shown in data S1.

For gene transfer to human EMO, Matrigel-maintaining EMO was depolymerized with the Cell Recovery Solution (Corning) at 4°C for 30 min. The human EMO were fragmented by repeated manual pipetting and centrifuged at 500*g* for 5 min. After rinsing with PBS, TrypLE Express was added to human EMO pellets and incubated at 37°C for 15 min. After adding DMEM/F-12 containing 10% FBS, EMO were dissociated into single cells via pipetting 50 times. Subsequently, lentivirus particles (20 μl) with polybrene (10 μM) were added, mixed with EMO using a pipette, transferred to a 48-well plate, and then incubated at 37°C for 5 min. Subsequently, the plate was sealed with parafilm and centrifuged at 300*g* for 60 min at room temperature for spin inoculation. After centrifugation, the supernatant was discarded without disrupting the cell pellet. After washing with PBS, the cells were resuspended in Matrigel and cultured in an EMO medium. After growing a sufficient number of EMO, single-cell treatment was performed in the same manner as described above, and EGFP^+^ cells were sorted using a FACSAria II cell sorter (BD Biosciences) and subjected to subsequent organoid culture. Gene transfer to human eSC was performed by adding lentiviruses to human eSC seeded in six-well plates.

The Tet-On 3G transactivator of the pTetOne vector (Takara) was PCR-amplified and cloned into the multi-cloning site of CS-CA-MCS. The resulting vector was designated as pCS-CA-Tet3G. The CAG promoter of CS-CA-MCS was replaced with the TRE3Gs promoter of pTetOne. The resulting vector was designated as pCS-3G (*74*). *KuO* was PCR-amplified from KuO-N1 (a gift from M. Davidson and A. Miyawaki; Addgene, plasmid no. 54793; http://n2t.net/addgene:54793) and cloned into pCS-3G to generate pCS-3G-KuO. Lentiviruses expressing Tet-On 3G and KuO were generated using pCS-CA-Tet3G and pCS-3G-KuO vectors, respectively. To establish KuO^+^ naïve human ESC, Tet-on 3G- and KuO-expressing lentiviruses were added to the culture medium of primed human ESC and selected manually on the basis of their fluorescence with doxycycline (100 ng/ml). Then, KuO^+^-primed human ESC were converted to naïve human ESC as described above. The primers used in this study are shown in data S1.

### Generation of blastoids

Blastoids were generated according to previous methods (*20*–*22*). Although we confirmed that the method of Yanagida *et al.* (*21*) and Kagawa *et al.* (*22*) could also produce blastoids, we used the method of Yu *et al.* (*20*) based on the formation efficiency in our environment and the cell line that we used. Naïve human ESC were dissociated with Accutase, and feeder cells were removed by seeding onto gelatin-coated dishes and incubated for 45 to 60 min at 37°C. Non-adherent human ESC were collected, and 30 to 50 cells per microwell were seeded onto 2% agarose-containing microwells fabricated using 3D petri dishes (Microtissues). The medium was replaced with hypoblast differentiation medium (HDM) (day 1). On day 3, the medium was replaced with trophoblast differentiation medium (TDM). Fresh TDM was replaced every 2 days until blastoids were observed in the microwells.

### Coculturing of blastoids and AO-EMO

The AO-EMO were transferred to 96-well low-attachment EZ-Bind Shut II plates (IWAKI). Then, blastoids (100 to 200 μm in diameter), having a cyst and an ICM-like structure inside, were picked up with a mouth pipette and placed in a well containing AO-EMO. Coculture was conducted under conditions on the basis of a mIVC culture (*40*). On days 0 and 1 of coculture, mIVC1 containing LIF (50 ng/ml; FUJIFILM Wako), EGF (50 ng/ml; FUJIFILM Wako), FGF2 (50 ng/ml; FUJIFILM Wako), heparin (1 μg/ml; FUJIFILM Wako), and Y27632 (10 μM; FUJIFILM Wako) were used. On day 2 of coculture, mIVC2 containing EGF (50 ng/ml), FGF2 (50 ng/ml), IGF-1 (20 ng/ml), BMP4 (40 ng/ml), heparin (1 μg/ml; FUJIFILM Wako), and 10% Matrigel was used. Half of the medium was replaced each day. All cocultures were performed under hypoxic conditions (5% O_2_). The composition of mIVC1 and mIVC2 media was as follows: mIVC1: advanced DMEM/F-12 (Thermo Fischer Scientific) supplemented with 20% FBS, 2 mM GlutaMAX, 1% penicillin/streptomycin, 1% ITS-X, 0.22% sodium lactate (Sigma-Aldrich), 8 nM β-estradiol, progesterone (200 ng/ml), and 25 μM *N*-acetyl-l-cysteine; and mIVC2: advanced DMEM/F-12 (Thermo Fischer Scientific) supplemented with 30% KSR, 2 mM GlutaMAX, 1% penicillin/streptomycin, 1% ITS-X, 0.22% sodium lactate, 8 nM β-estradiol, progesterone (200 ng/ml), and 25 μM *N*-acetyl-l-cysteine. Coculture was conducted at 37°C in a 5% CO_2_ and 5% O_2_ condition. The rate of blastoid attachment was quantified as the ratio of the number of blastoids that remained attached after washing and fixation following coculture to the number of blastoids that were initially added.

### DNA-FISH

Cells were cultured on collagen-coated coverslips (MATSUNAMI Glass) and fixed in 4% PFA (FUJIFILM Wako) at room temperature for 20 min. After being washed with PBS and 70% ethanol, coverslips were permeabilized in PBS with 0.4% Triton X-100 at 4°C for 7 min. For antigen retrieval, coverslips were heated at 90°C in HistoVT one (Nacalai Tesque) for 30 min and subsequently denatured in 2× saline sodium phosphate EDTA (SSPE) (DOJINDO) with 70% formamide at 73°C for 5 min. Coverslips were dehydrated in 70, 85, and 100% ethanol and air-dried. Human XY chromosome FISH probe (Chromosome Science Labo Inc.) was applied and hybridized with DNA by heating at 80°C for 20 s followed by incubation at 37°C overnight. Coverslips were washed with 2× SSPE and incubated in 2× SSPE with 50% formamide at 37°C for 20 min. After a wash in 1× SSPE, coverslips were incubated with the CanGetSignal immunostain Solution B (TOYOBO) containing fluorescein isothiocyanate and biotin antibodies (table S1) at 37°C for 1 hour. Coverslips were washed in 4× SSPE with 0.05% Tween 20 (Bio-Rad) and in 4× SSPE and subsequently incubated in the CanGetSignal immunostain Solution B containing Alexa Fluor 488– and Alexa Fluor 555–cojugated secondary antibodies at 37°C for 1 hour. After being washed in 4× SSPE with 0.05% Tween 20 and in 4× SSPE, coverslips were stained with Hoechst 33342 and Phalloidin-iFluor 647 reagent (Abcam).

### Coculturing of human blastoids and human eSC

For 3D coculture, EGFP-eSC were embedded in a collagen/Matrigel mix (7:3) using μ-Slide Angiogenesis (ibidi) at a density of 5 × 10^6^ to 1 × 10^7^ cells/ml. Coculture was conducted in mIVC1 medium supplemented with 50 μM 8-Br-cAMP. On days 1 to 3 of coculture, the cells were fixed with 4% PFA for 30 min at room temperature and processed for whole-mount staining as described above.

The length of the CGB^+^ was determined by measuring the distance and fluorescence intensity of EGFP and CGB in each focal plane. To achieve this, a line was drawn through the center of the blastoid in a random direction, and the fluorescence intensity was measured along the line using ZEN software (Carl Zeiss). The threshold was set on the basis of CGB localization. The distance of the CGB^+^ line was calculated by dividing it by whether it colocalized with EGFP.

To quantify the percentage of cells fused with stromal cells, immunostaining images captured with a confocal microscope (LSM780) were used to count the cells merged with EGFP for CGB^+^ and OCT4^+^ cells, and the percentage was calculated.

One or 2 days before 2D coculture, EGFP-eSC were seeded at a cell density of 5 × 10^4^ cells per well in ibidi eight-well μ-Slides (ibidi) with MEF medium containing 1 μM MPA and 50 μM 8-Br-cAMP. Human blastoids were manually picked using a mouth pipette and seeded onto eSC. Coculture was conducted using mIVC1 supplemented with 50 μM 8-Br-cAMP until day 2. On day 2, the medium was replaced with mIVC2 supplemented with 50 μM 8-Br-cAMP. On day 3 of coculture, the cells were fixed with 4% PFA. The blastoid area was quantified by drawing a line along the shape of the adhered blastoids and quantifying the area using the Fiji software.

A split GFP system (*49*) was used to confirm cell fusion. GFP1-10 and GFP11-tagged histone H2B were synthesized and cloned into the CS-CA-MCS plasmid, yielding pCS-CA-GFP1-10 and pCS-CA-GFP11-H2B constructs, respectively. Lentivirus vectors carrying either GFP1-10 or GFP11-H2B were generated as previously described in this section. The oligo sequences used for vector construction are provided in data S1.

### Human blastocyst culture

Human blastocysts were thawed using the Cryotop Safety Kit Thawing Kit (Kitazato). Frozen-thawed human blastocysts (5 to 6 days after fertilization) were treated with Tyrode’s solution to remove the zona pellucida. Thereafter, blastocysts were cultured as described above in the “Coculturing of blastoids and AO-EMO” or “Coculturing of human blastoids and human eSC” section. In both cases, after coculturing, the blastocysts were cultured for no more than 5 days before being fixed and used for analysis.

### 2D culture of human EMO

Human EMO were collected and dissociated with TrypLE Express with Y27632 (10 μM) for 15 min at 37°C. The cells were then seeded onto type I collagen–coated 60-mm dish (IWAKI) or Matrigel-coated (5 μg/cm^2^) dishes. For coating, 15 μl of Matrigel was added to 3 ml of DMEM/F-12 and incubated at room temperature for 1 hour. Cells were further cultured in an ECSY medium. The cells were fixed with 4% PFA at room temperature for 30 min for rhodamine staining. After washing with deionized water, a 2% rhodamine B (Muto Pure Chemicals) solution was added and incubated overnight at room temperature. The stained cells were washed with 0.2 M HCl and dried.

### Flow cytometry

The human eSC were dissociated using TrypLE Express and resuspended in PBS containing 1% BSA. The cells were stained with antibodies against CD73 (BioLegend, no. 344004), CD90 (eBioscience, no. 11-0909-42), and CD31 (Abcam, no. ab134168) for 30 to 60 min on ice. A FACSCanto II (BD Biosciences) was used for the analysis.

### In vitro decidualization of human eSC

Human eSC were seeded at a density of 2.8 × 10^4^ cells per well in 12-well plates and cultured in 10% FBS containing DMEM/F-12 (FUJIFILM Wako, no. 048-29785). On day 4, the medium was switched to in vitro decidualization medium (IVDM), and cells were cultured for an additional 3 days. IVDM comprised phenol red–free DMEM/F-12 (Gibco) with 2% heat-inactivated charcoal-stripped FBS (Thermo Fisher Scientific), 1% penicillin/streptomycin, 10 nM β-estradiol (E_2_), 1 μM MPA, and 50 μM 8-Br-cAMP. The control medium comprised IVDM lacking E_2_, MPA, and 8-Br-cAMP.

### Ligand-receptor analysis

snRNA-seq data were sourced from our AO-EMO dataset. For the elucidation of cellular communication patterns, we used the CellChat tool (version 1.6.1) (*75*). The CellChatDB.human integrated with “Secreted Signaling” from the CellChatDB was used to infer communication probabilities, allowing us to discern potential ligand-receptor candidates. Visual representation of the communication networks was achieved through the netVisual_chord_gene function, designating epithelial cells as the source (sources.use) and both stromal and endothelial cells as the targets (targets.use).

### Statistical analysis

Statistical analysis was performed using JMP software (SAS Institute, Cary, NC, USA) or R (version 4.4.4). The Shapiro-Wilk test or the Kolmogorov-Smirnov test was used to examine the normality of the data distribution. Leven’s test was used to evaluate the equality of variances. If the data were normally distributed and had equal variances, then the Student’s *t* test was applied for comparisons between independent groups. If the data were not normally distributed or had unequal variances, then the Welch’s *t* test was used. Multiple comparisons across various groups were performed using the Mann-Whitney *U* test, and adjustments for multiple comparisons were made using the Benjamini-Hochberg procedure. A *P* value of <0.05 was considered statistically significant. All error bars represent means ± SD.
