# Supplementary material for: Modeling embryo-endometrial interface recapitulating human embryo implantation
Source: Sci Adv. 2024 Feb 23;10(8):eadi4819. doi: 10.1126/sciadv.adi4819 (PMC10889356; doi:10.1126/sciadv.adi4819)
Supplement: Supplementary file 1 — Figs. S1 to S9 Table S1 Legends for data S1 to S3 [file sciadv.adi4819_sm.pdf]

Science Advances
AAAS

Supplementary Materials for

Modeling embryo-endometrial interface recapitulating human embryo implantation

Shun Shibata *et al.*

Corresponding author: Shun Shibata, shun.shibata.a3@tohoku.ac.jp; Takahiro Arima, tarima@med.tohoku.ac.jp

Sci. Adv. **10**, eadi4819 (2024)
DOI: 10.1126/sciadv.adi4819

The PDF file includes:

Figs. S1 to S9
Table S1
Legends for data S1 to S3

Other Supplementary Material for this manuscript includes the following:

Data S1 to S3

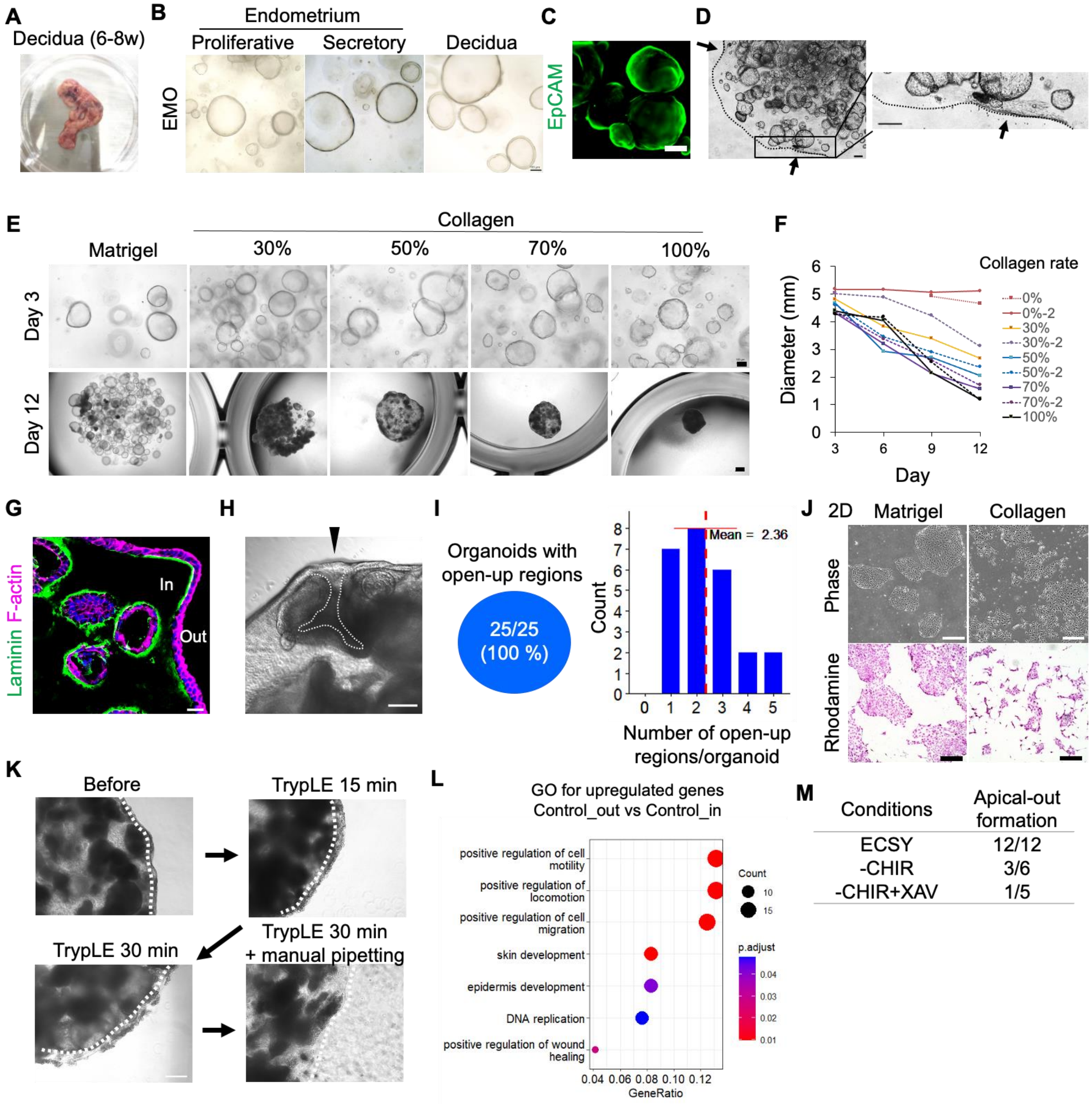

Conditions	Apical-out formation
ECSY	12/12
-CHIR	3/6
-CHIR+XAV	1/5

Fig. S1. Generation of apical-out endometrial organoids (AO-EMO). (**A**) Human decidua (6–9 weeks gestation). (**B**) Bright-field images of human endometrium- and decidua-derived EMO. Scale bar, 100 µm. (**C**) Whole-mount imaging of EMO stained for EpCAM. Scale bar, 500 µm. (**D**) Phase contrast images of EMO cultured in a collagen-based culture on day 3. Black arrows indicate areas where epithelial cells began to cover the surface and the collagen-based gel started to contract. Dashed lines outline the outer edge of the gel. Scale bars, 200 µm. (**E**) Bright-field images of EMO in Matrigel and 30%, 50%, 70%, and 100% collagen-based gels on days 3 and 12 of culture. Scale bars, 100 µm (upper) and 500 µm (lower). (**F**) Quantification of diameters of EMO or gel. (**G**) Immunostaining image of AO-EMO stained for laminin (green) and F-actin (magenta); nuclei are stained with Hoechst (blue). Scale bar, 20 µm. (**H**) Phase contrast image of the open-up region in AO-EMO. The black arrowhead indicates the open-up region. The dotted

line traces the outline of the inside of the endometrial epithelium. Scale bar, 100 µm. (**I**) Quantification of organoids that possess open-up regions (left) and the number and distribution of open-up regions per organoid. The vertical red dashed line indicates the mean value, while the horizontal red lines represent the standard deviation. (**J**) Phase contrast and rhodamine staining images of 2D-cultured human EMO on Matrigel- or collagen-coated dishes. Scale bars, 500 µm (upper) and 200 µm (lower). (**K**) Phase contrast images of AO-EMO during the separation process. Scale bar, 200 µm. (**L**) GO terms related to biological processes enriched in outer (Control_out) versus inner (Control_in) cells of AO-EMO pre-treated with hormone. (**M**) Quantification of AO formation rate under various culture conditions.

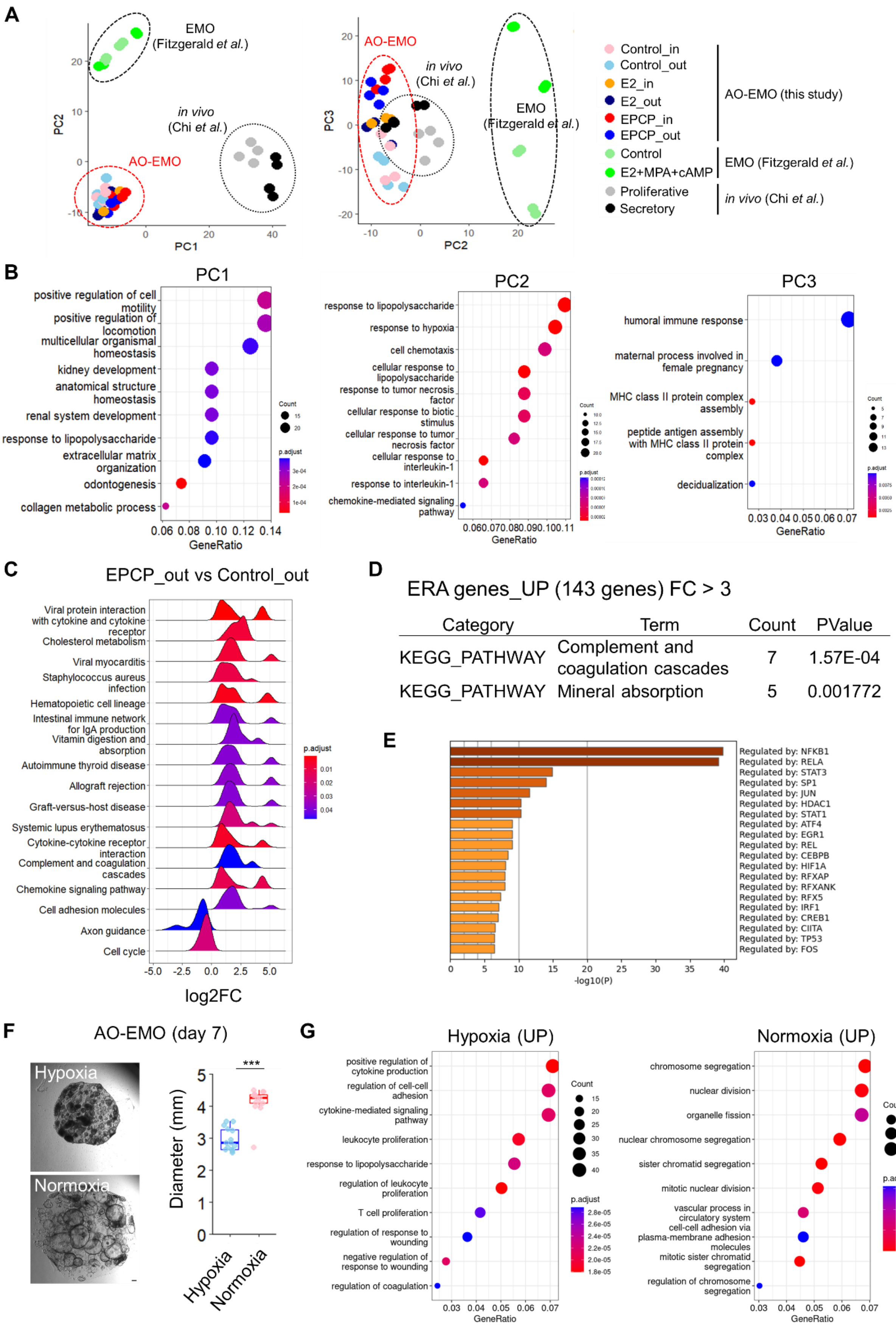

A
EMO (Fitzgerald et al.)
in vivo (Chi et al.)
AO-EMO
PC1
PC2
PC3
Control_in
Control_out
E2_in
E2_out
EPCP_in
EPCP_out
AO-EMO (this study)
Control
E2+MPA+cAMP
EMO (Fitzgerald et al.)
Proliferative
Secretory
in vivo (Chi et al.)
B
PC1
positive regulation of cell motility
positive regulation of locomotion
multicellular organismal homeostasis
kidney development
anatomical structure homeostasis
renal system development
response to lipopolysaccharide
extracellular matrix organization
odontogenesis
collagen metabolic process
GeneRatio
Count
p.adjust
PC2
response to lipopolysaccharide
response to hypoxia
cell chemotaxis
cellular response to lipopolysaccharide
response to tumor necrosis factor
cellular response to biotic stimulus
cellular response to tumor necrosis factor
cellular response to interleukin-1
response to interleukin-1
chemokine-mediated signaling pathway
PC3
humoral immune response
maternal process involved in female pregnancy
MHC class II protein complex assembly
peptide antigen assembly with MHC class II protein complex
decidualization
C
EPCP_out vs Control_out
Viral protein interaction with cytokine and cytokine receptor
Cholesterol metabolism
Viral myocarditis
Staphylococcus aureus infection
Hematopoietic cell lineage
Intestinal immune network for IgA production
Vitamin digestion and absorption
Autoimmune thyroid disease
Allograft rejection
Graft-versus-host disease
Systemic lupus erythematosus
Cytokine-cytokine receptor interaction
Complement and coagulation cascades
Chemokine signaling pathway
Cell adhesion molecules
Axon guidance
Cell cycle
log2FC
p.adjust
D
ERA genes_UP (143 genes) FC > 3
Category | Term | Count | PValue
KEGG_PATHWAY | Complement and coagulation cascades | 7 | 1.57E-04
KEGG_PATHWAY | Mineral absorption | 5 | 0.001772
E
Regulated by: NFKB1
Regulated by: RELA
Regulated by: STAT3
Regulated by: SP1
Regulated by: JUN
Regulated by: HDAC1
Regulated by: STAT1
Regulated by: ATF4
Regulated by: EGR1
Regulated by: REL
Regulated by: CEBPB
Regulated by: HIF1A
Regulated by: RFXAP
Regulated by: RFXANK
Regulated by: RFX5
Regulated by: IRF1
Regulated by: CREB1
Regulated by: CIITA
Regulated by: TP53
Regulated by: FOS
-log10(P)
F
AO-EMO (day 7)
Hypoxia
Normoxia
Diameter (mm)
***
G
Hypoxia (UP)
positive regulation of cytokine production
regulation of cell-cell adhesion
cytokine-mediated signaling pathway
leukocyte proliferation
response to lipopolysaccharide
regulation of leukocyte proliferation
T cell proliferation
regulation of response to wounding
negative regulation of response to wounding
regulation of coagulation
Normoxia (UP)
chromosome segregation
nuclear division
organelle fission
nuclear chromosome segregation
sister chromatid segregation
mitotic nuclear division
vascular process in circulatory system
cell-cell adhesion via plasma-membrane adhesion molecules
mitotic sister chromatid segregation
regulation of chromosome segregation

Fig. S2. Collagen-based culture enhances the maturation and spatial heterogeneity of endometrial epithelial cells.

(**A**) Principal component analysis of the transcriptome of AO-EMO, conventional EMO (*31*), and *in vivo* endometrial epithelial cells (*33*). (**B**) GO terms (biological processes) enriched in genes of PC1, PC2, and PC3. (**C**) Pre-ranked gene set enrichment analysis (GSEA) of upregulated genes in outer cells treated with hormone (EPCP_out) versus control (Control_out). (**D**) KEGG enrichment analysis of extracted upregulated genes (143 genes) in the listed Endometrial Receptivity Analysis (ERA) test (*36*). (**E**) Predicted upstream transcription factors using upregulated genes in EMO cultured in collagen. (**F**) Bright-field images (left) and diameters (right) of AO-EMO cultured under hypoxic and normoxic conditions on day 7 of culture. Scale bar, 200 µm. n = 15 AO-EMO. (**G**) GO terms related to biological processes enriched in upregulated genes in AO-EMO cultured under hypoxic (left) and normoxic (right) conditions.

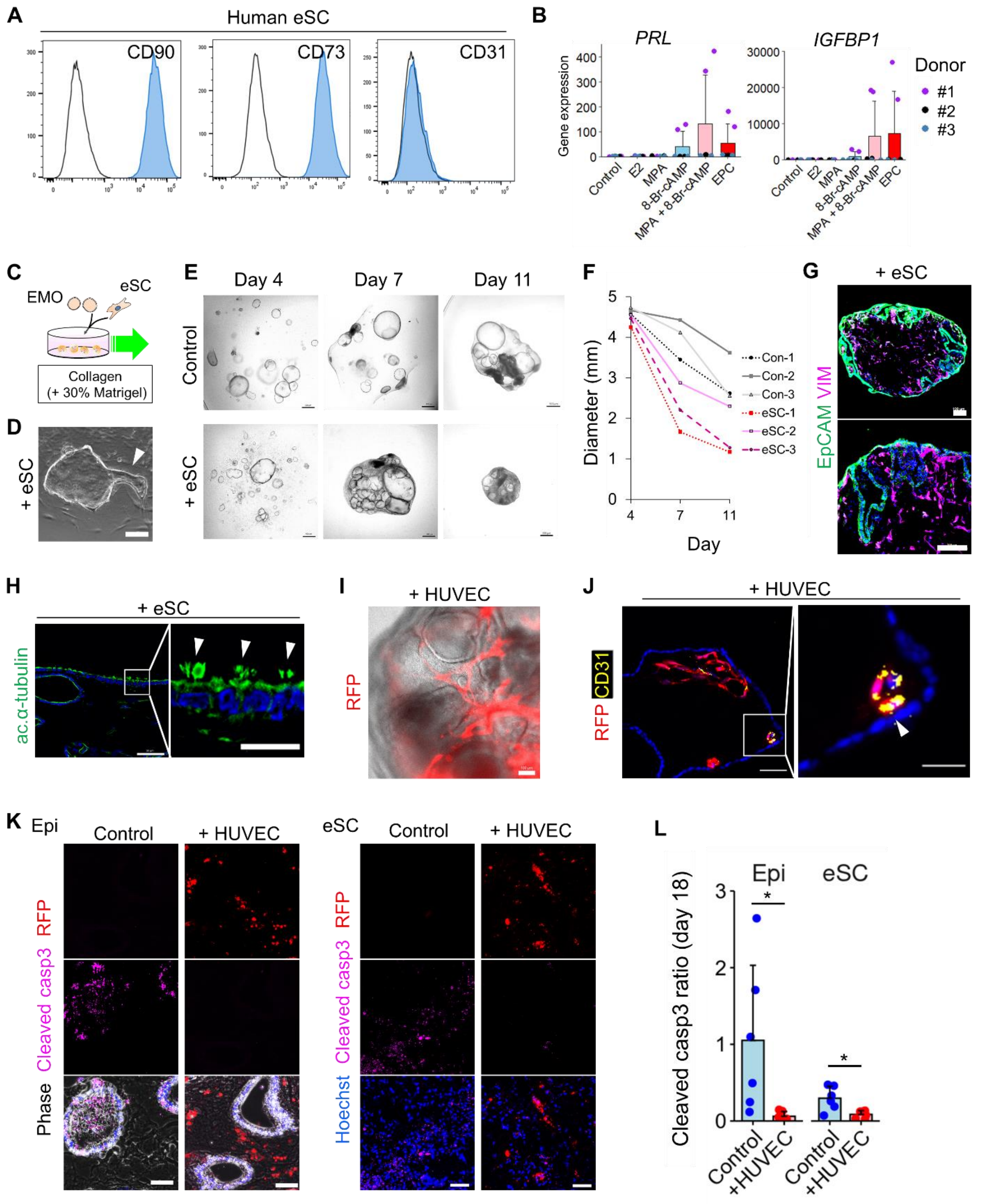

Fig. S3. Integrating stromal cells and vascular network into AO-EMO. (**A**) Flowcytometric analysis of human eSC with endometrial stromal (CD90 and CD73) and endothelial (CD31) markers. (**B**) Gene expression levels of eSC treated with vehicles (Control), E2, MPA, 8-Br-cAMP, MPA + 8-Br-cAMP, and E2 + MPA + 8-Br-cAMP (EPC). Data shown as mean ± SD; n

= 6 from three individual donors. (**C**) Schematic representation of the culture method. (**D**) Phase contrast image of EMO co-cultured with eSC. The arrowhead indicates the protrusion of EMO. Scale bar, 100 µm. (**E**) Bright-field images of EMO co-cultured with or without eSC in collagen-based gel on days 4, 7, and 11. Scale bars, 500 µm. (**F**) Quantification of diameters of EMO or gel of AO-EMO with or without eSC. (**G**) Fluorescence images of AO-EMO including eSC stained for EpCAM and VIM; nuclei stained with Hoechst (blue). Scale bars, 100 µm. (**H**) Fluorescence images of AO-EMO including eSC stained for acetylated (ac.) α-tubulin; nuclei are stained with Hoechst (blue). Scale bars, 50 µm (upper) and 20 µm (lower). Arrowheads indicate ac. α-tubulin^{+} cilia. (**H**) Bright-field and fluorescence image of AO-EMO, including RFP-HUVEC. Scale bar, 500 µm. (**I**) Bright-field and fluorescence image of AO-EMO, including eSC and RFP-HUVEC. Scale bar, 500 µm. (**J**) Fluorescence images of AO-EMO including RFP-HUVEC stained for CD31; nuclei stained with Hoechst (blue). Arrowheads indicate luminal structures. Scale bars, 50 µm and 20 µm (magnified). (**K**) Phase contrast and fluorescence images of AO-EMO co-cultured with RFP-HUVEC (red) and AO-EMO + eSC co-cultured with RFP-HUVEC (red), cultured for 18 days stained for cleaved caspase (Casp)-3; nuclei stained with Hoechst (blue). Scale bars, 50 µm. (**L**) Quantification of cleaved Casp3^{+} ratio normalized to nuclei. Data shown as mean ± SD; $^{*}P < 0.05$; n = 6 frozen sections from three independent experiments.

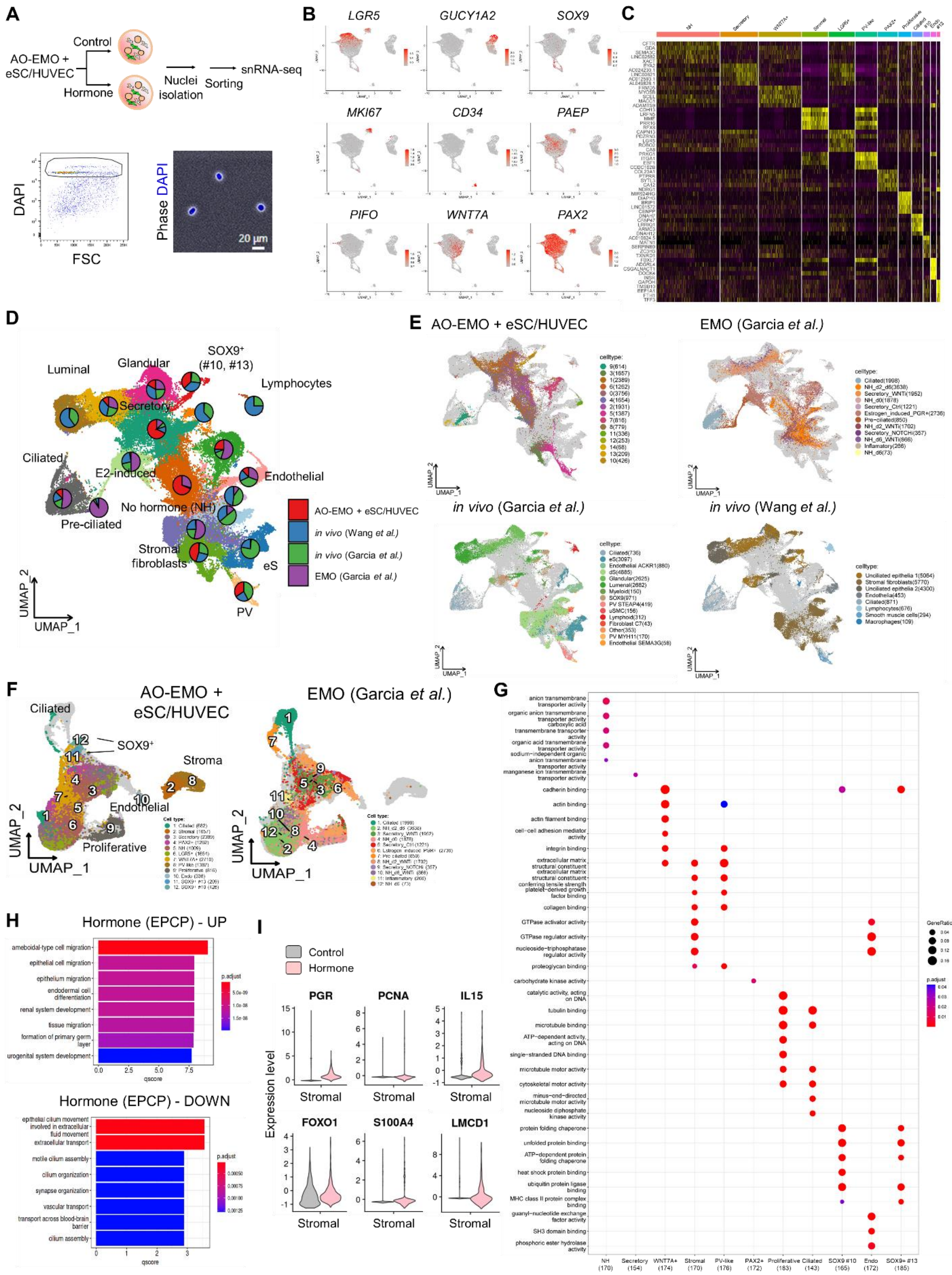
A
Control
AO-EMO +
eSC/HUVEC
Hormone
Nuclei
isolation
Sorting
snRNA-seq
DAPI
FSC
Phase DAPI
20 μm
B
LGR5
GUCY1A2
SOX9
MKI67
CD34
PAEP
PIFO
WNT7A
PAX2
C
D
Luminal
Glandular
SOX9⁺
(#10, #13)
Lymphocytes
Secretory
Ciliated
E2-induced
Endothelial
No hormone (NH)
Pre-ciliated
Stromal
fibroblasts
eS
PV
AO-EMO + eSC/HUVEC
in vivo (Wang et al.)
in vivo (Garcia et al.)
EMO (Garcia et al.)
UMAP_2
UMAP_1
E
AO-EMO + eSC/HUVEC
EMO (Garcia et al.)
in vivo (Garcia et al.)
in vivo (Wang et al.)
F
Ciliated
AO-EMO +
eSC/HUVEC
SOX9⁺
Stroma
Endothelial
Proliferative
EMO (Garcia et al.)
UMAP_2
UMAP_1
G
cadherin binding
actin binding
actin filament binding
integrin binding
collagen binding
GTPase activator activity
GTPase regulator activity
proteoglycan binding
carbohydrate kinase activity
tubulin binding
microtubule binding
single-stranded DNA binding
microtubule motor activity
cytoskeletal motor activity
protein folding chaperone
unfolded protein binding
heat shock protein binding
SH3 domain binding
NH
Secretory
WNT7A+
Stromal
PV-like
PAX2+
Proliferative
Ciliated
SOX9 #10
Endo
SOX9+ #13
H
Hormone (EPCP) - UP
ameboidal-type cell migration
epithelial cell migration
epithelium migration
endodermal cell differentiation
renal system development
tissue migration
formation of primary germ layer
urogenital system development
qscore
Hormone (EPCP) - DOWN
motile cilium assembly
cilium organization
synapse organization
vascular transport
cilium assembly
qscore
I
Control
Hormone
PGR
PCNA
IL15
FOXO1
S100A4
LMCD1
Expression level
Stromal

Fig. S4. Characterization of AO-EMO at the single-cell level. (**A**) Schematic representation of the experimental procedure (upper). Flow cytometry image for DAPI^{+} sorting of AO-EMO + eSC/HUVEC, and phase contrast and fluorescence images of sorted nuclei (lower); Scale bar, 20 µm. (**B**) Feature plots showing expression levels and distribution of marker genes. (**C**) Heatmap showing the top five most upregulated genes in each cluster of AO-EMO + eSC/HUVEC. (**D and E**) UMAP projection of the four datasets integration (*30*, *35*). The pie charts show the proportion of each cell type occupied by each dataset (D). Each of the four datasets is overlaid with a unique color on the integrated data (E). (**F**) UMAP plot representing the integrated data from AO-EMO + eSC/HUVEC and EMO (Garcia *et al.*). (**G**) Dot plot showing enriched GO terms in each cluster of AO-EMO + eSC/HUVEC. (**H**) Bar graph representing GO term (BP) enriched in AO-EMO + eSC/HUVEC with (upper) or without (lower) hormone (EPCP) treatment. (**I**) Violin plots representing the expression levels of hormone-responsive genes of the stromal population in AO-EMO + eSC/HUVEC with or without hormone treatment.

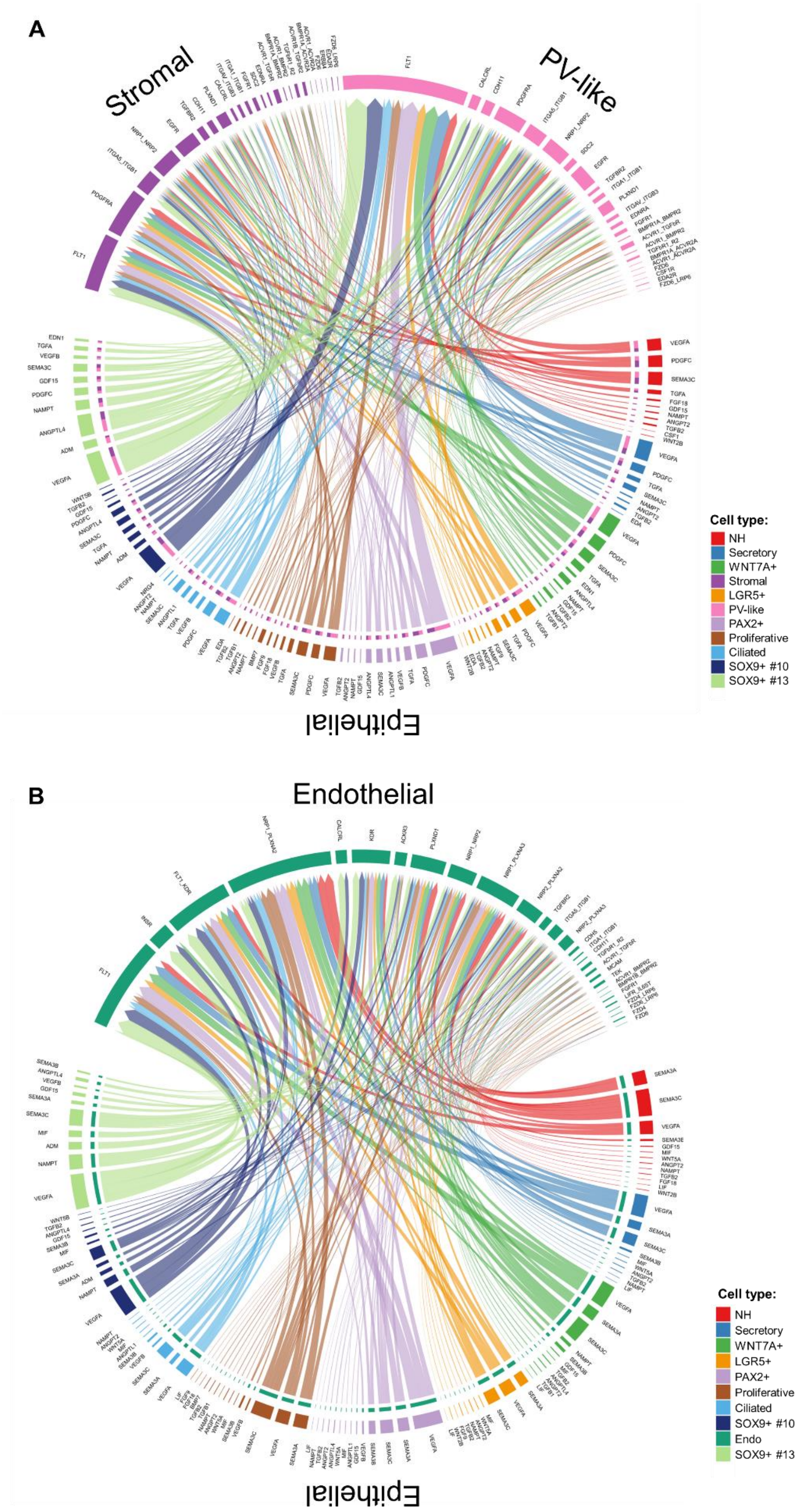
A
Stromal
PV-like
Epithelial
Cell type:
NH
Secretory
WNT7A+
Stromal
LGR5+
PV-like
PAX2+
Proliferative
Ciliated
SOX9+ #10
SOX9+ #13
B
Endothelial
Epithelial
Cell type:
NH
Secretory
WNT7A+
LGR5+
PAX2+
Proliferative
Ciliated
SOX9+ #10
Endo
SOX9+ #13

Fig. S5. Ligand-receptor analysis in AO-EMO + eSC/HUVEC snRNA-seq data. This visualization represents inferred cellular communication patterns between different cell types based on ligand-receptor interactions. (**A**) Potential interactions between epithelial and endothelial cells, while (**B**) highlights those between epithelial and stromal cells.

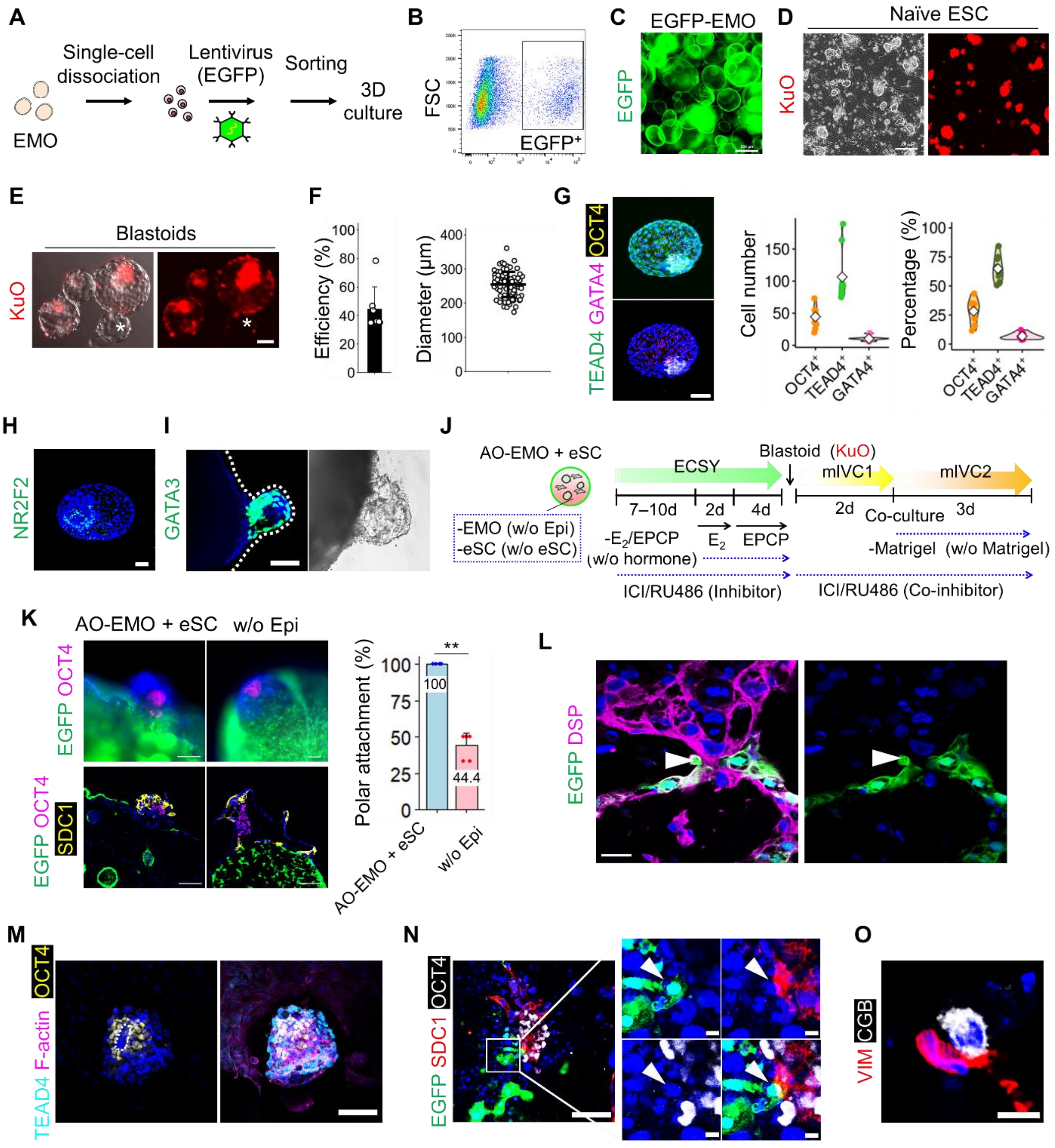

Fig. S6. Feto–maternal assembloids mimic human embryo implantation. (**A**) Schematic representation of the experimental procedure for generating EGFP-expressing human EMO (EGFP-EMO). (**B**) Flow cytometry graph for EGFP$^+$ sorting of lentivirus-infected EMO. (**C**) Fluorescence image of EGFP-EMO. Scale bar, 500 μm. (**D**) Phase contrast and fluorescence (KuO: red) images of naïve human ESC. Scale bar, 200 μm. (**E**) Bright-field and fluorescence images of KuO$^+$ human blastoids. Scale bar, 100 μm. * indicates blastoid lacking ICM-like cells. (**F**) Quantification of formation efficiency (left) and diameter (right) of blastoids. n = 6 independent induction experiments (left) and 85 blastoids (right). (**G**) Whole-mount imaging of human blastoid stained for TEAD4, GATA4, and OCT4 (left). Scale bar, 100 μm. Quantification

of the $OCT4^+$, $TEAD4^+$, and $GATA4^+$ cells, and their respective proportions of the total cell number in blastoids (right). n = 9 blastoids. (**H**) Whole-mount imaging of human blastoid stained for NR2F2; nuclei stained with Hoechst (blue). Scale bar, 100 μm. (**I**) Bright-field and whole-mount imaging of attached blastoid stained for GATA3; nuclei stained with Hoechst (blue). Scale bar, 100 μm. (**J**) Schematic representation of the experimental procedure for quantifying the adhesion rate of blastoids in various culture conditions. (**K**) Whole-mount imaging (upper) and fluorescence images of frozen sections (lower) of feto–maternal assembloid (Epi, $EGFP^+$ [AO-EMO + eSC]; eSC, $EGFP^+$ [w/o Epi]) stained for OCT4 and SDC1; nuclei stained with Hoechst (blue). Scale bars, 100 μm (left). Quantification of the ratio of polar attachment of blastoids (right). n = 7 (AO-EMO + eSC) and 6 (w/o Epi) independent co-culture experiments. $^{**}P < 0.01$. (**L**) Fluorescence images of feto–maternal assembloid stained for Desmoplakin (DSP); nuclei stained with Hoechst (blue). Scale bar, 20 μm. Arrowheads indicate invading blastoids. (**M** and **N**) Whole-mount imaging of feto–maternal assembloid (Epi: $EGFP^+$) stained for OCT4 and TEAD4 (M) or SDC1 (N); nuclei stained with Hoechst (blue). Arrowheads indicate $EGFP^+$/$SDC1^+$ cells. Scale bars, 100 μm and 10 μm (magnified). (**O**) Fluorescence images of feto–maternal assembloid stained for VIM and CGB; nuclei stained with Hoechst (blue). Scale bar, 10 μm.

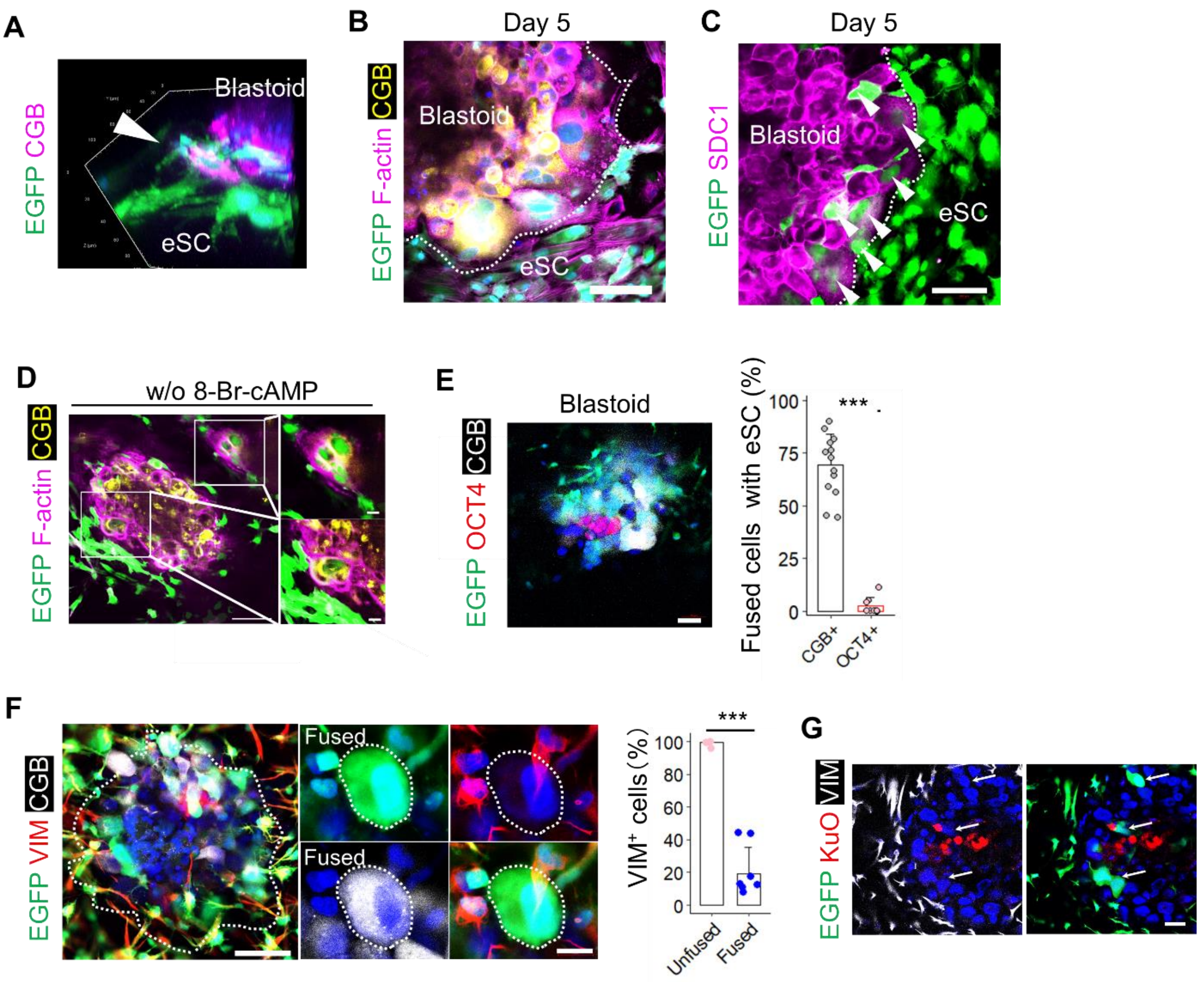

Fig. S7. The invading syncytium fuses with endometrial stromal cells. (**A**) Reconstructed 3D image of EGFP+/CGB+ cells at the blastoid–eSC interface. (**B**) Whole-mount imaging of blastoids co-cultured with eSC (EGFP+) on day 5 stained for CGB and F-actin; nuclei stained with Hoechst (blue). Scale bar, 100 µm. (**C**) Whole-mount imaging of blastoids co-cultured with eSC (EGFP+) on day 5 stained for SDC1; nuclei stained with Hoechst (blue). Scale bar, 100 µm. Arrowheads point to EGFP+ nuclei within SDC1+ cells. The dashed line delineates the interface between the blastoid and eSC regions. (**D**) Whole-mount imaging of blastoid co-cultured with eSC (EGFP+) without 8-Br-cAMP stained for F-actin and CGB; nuclei stained with Hoechst (blue). Scale bars, 100 µm and 20 µm (magnified). (**E**) Whole-mount imaging of blastoid co-cultured with eSC (EGFP+) stained for OCT4 and CGB (left). Quantification of fused cells with eSC (right); n = 13 blastoids of two independent experiments; ***$P < 0.001$. (**F**) Whole-mount imaging of blastoid co-cultured with eSC (EGFP+) in a collagen-based gel on day 3 stained for VIM and CGB; nuclei stained with Hoechst (blue). Scale bars, 100 µm and 20 µm (magnified). The dashed lines delineate fused cells. (**G**) Whole-mount imaging of blastoid (KuO+) co-cultured with eSC (EGFP+) stained for VIM; nuclei stained with Hoechst (blue). Arrows point out to EGFP+/VIM- cells. Scale bar, 50 µm.

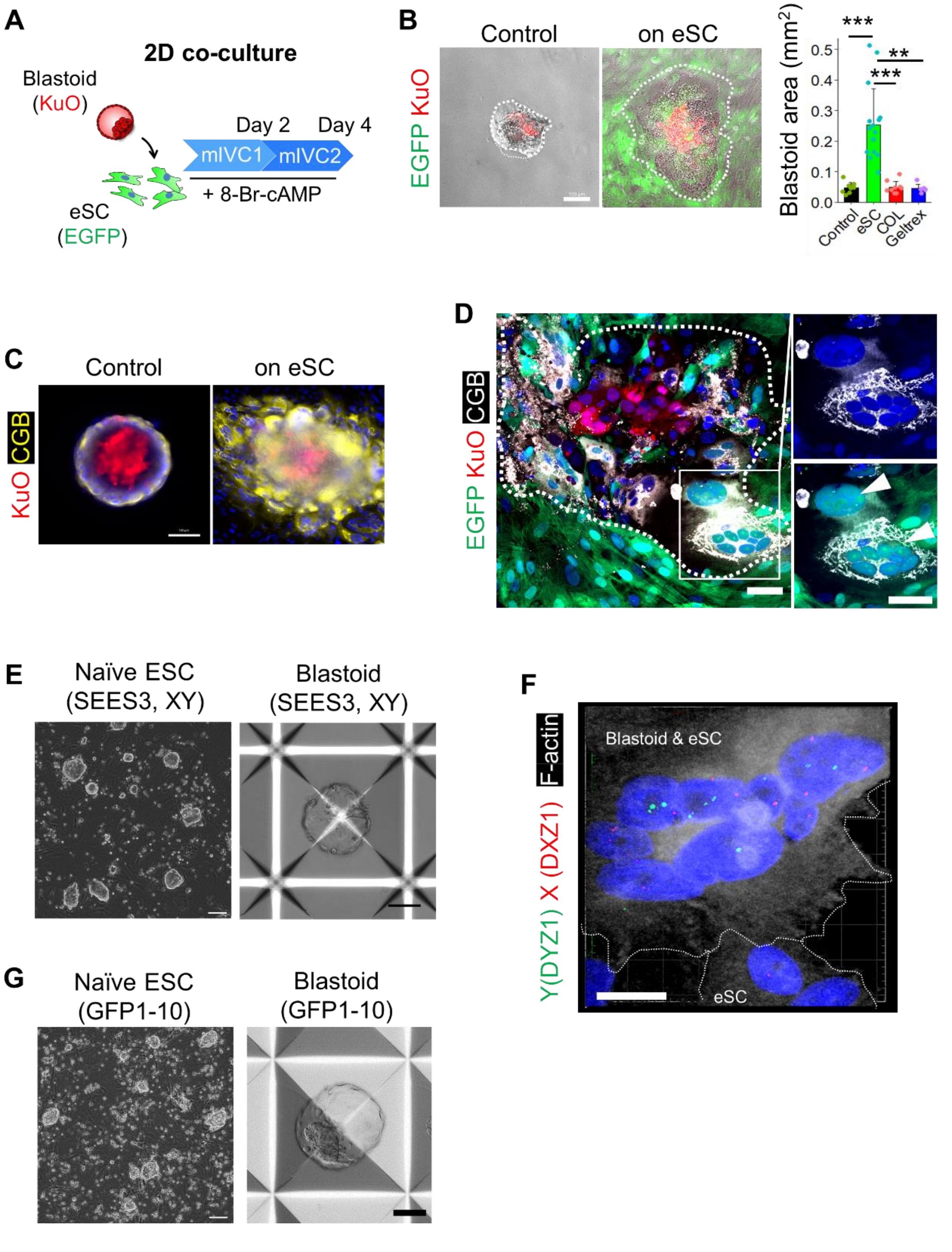

Fig. S8. 2D co-culture of blastoids and eSC. (**A**) Schematic illustration of 2D co-culture experiment. (**B**) Phase contrast and fluorescence images of adhered blastoids (KuO^+) with or without eSC ($EGFP^+$) (left). Scale bar, 100 µm. Quantification of adhered-blastoid area on culture plate (Control), eSC, type I collagen-coated dish (COL), and Geltrex-coated dish (Geltrex) (right); Data shown as mean ± SD; ***$P < 0.001$, **$P < 0.01$; n = 9 (Control), 16 (eSC), 10 (COL), and 6 (Geltrex) blastoids. (**C**) Fluorescence images of adhered blastoids

(KuO^+) on culture plate (Control) or eSC (on eSC) stained for CGB; nuclei stained with Hoechst (blue). Scale bar, 100 µm. (**D**) Fluorescence images of adhered blastoid (KuO^+) on eSC ($EGFP^+$) stained for CGB; nuclei stained with Hoechst (blue). Arrowheads indicate CGB^+ cells incorporating $EGFP^+$ eSC nuclei. Scale bars, 50 µm. (**E**) Phase contrast image of naïve ESC (SEES3 line) and bright-field image of SEES3-derived blastoid. Scale bars, 100 µm. (**F**) X (DXZ1) and Y (DYZ1) chromosome detection of multi-nucleated cells in blastoid (SEES3-derived) co-cultured with eSC stained for F-actin. Maximum projection of fluorescence image; nuclei stained with Hoechst (blue). Scale bar, 20 µm. The dashed lines delineate the outlines of the cells based on the cytoplasm stained for F-actin. (**G**) Phase contrast image of naïve ESC and bright-field image of blastoid, both expressing GFP1-10. Scale bars, 100 µm.

A

	Adhesion to AO-EMO
Human blastocyst	2/14

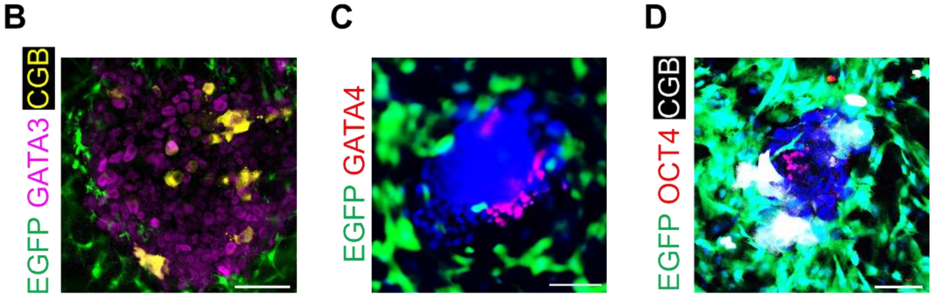

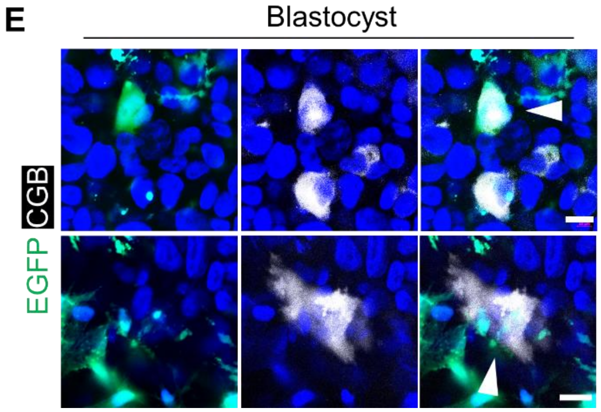

Fig. S9. Culture and analysis of human blastocysts for investigating interaction with endometrial cells. (**A**) Quantification of attached human blastocysts. (**B** to **D**) Whole-mount imaging of human blastocyst co-cultured with eSC ($EGFP^+$) in collagen-based gel on day 3 stained for GATA3, CGB (B), GATA4 (C), and OCT4, CGB (D); nuclei stained with Hoechst (blue). Scale bars, 100 µm. (**E**) Whole-mount imaging of human blastocyst co-cultured with eSC ($EGFP^+$) in collagen-based gel on day 3 stained for CGB; nuclei stained with Hoechst (blue). Arrowheads indicate CGB^+/$EGFP^+$ fused cells. Scale bars, 20 µm.

Table S1. Antibodies used in this study.

Antibody	Supplier	Cat#
Mouse anti-EpCAM	Cell Signaling	Cat#2929S
Rabbit anti-laminin	Abcam	Cat#ab11575
Rabbit anti-SOX9	Atlas antibodies	Cat#HPA001758
Rabbit anti-PGR	Cell Signaling	Cat#8757S
Rabbit anti-vimentin	Cell Signaling	Cat#5741S
Mouse anti-acetylated α-tubulin	Sigma	Cat#T7451-25UL
Rabbit anti-cleaved caspase 3	Cell Signaling	Cat#9661T
Mouse anti-SDC1 (PE-conjugated)	Miltenyi Biotec	Cat#130-081-301
Rabbit anti-Oct4A	Cell Signaling	Cat#2840S
Mouse anti-CGB	Abcam	Cat#ab9582
Rabbit anti-hCG	Dako	Cat#IS508
Rat anti-GATA4	Thermo Fisher	Cat#14-9980-82
Rabbit anti-NR2F2	Abcam	Cat#ab211776
Rabbit anti-CD31	Abcam	Cat#ab134168
Rabbit anti-Desmoplakin (DSP)	Proteintech	Cat#23518-1-AP
Mouse anti-CD73 (PE-conjugated)	BioLegend	Cat#344004
Mouse anti-CD90 (FITC-conjugated)	eBioscience	Cat#11-0909-42
Goat anti-rabbit IgG (Alexa Fluor 488-conjugated)	Cell Signaling	Cat#4412
Goat anti-mouse IgG (Alexa Fluor 488-conjugated)	Cell Signaling	Cat#4408
Goat anti-rabbit IgG (Alexa Fluor 555-conjugated)	Cell Signaling	Cat#4413
Goat anti-mouse IgG (Alexa Fluor 555-conjugated)	Cell Signaling	Cat#4409
Goat anti-rabbit IgG (Alexa Fluor 647-conjugated)	Cell Signaling	Cat#4409
Goat anti-mouse IgG (Alexa Fluor 647-conjugated)	Cell Signaling	Cat#4409
Rabbit anti-FITC	ThermoFisher	Cat#71-1900
Mouse anti-Biotin	Abcam	Cat#ab201341

Data S1. (separate file)
Oligonucleotides used in this study.

Data S2. (separate file)
RNA-seq sample information and processed data.

Data S3. (separate file)
snRNA-seq sample information and differentially expressed genes (DEGs) list.
